# Enhancing Anode-Free Battery Performance with Self-Healing Single-Ion Conducting PAMPS-*co*-PBA Copolymer Interfaces

**DOI:** 10.1021/acsami.4c22501

**Published:** 2025-03-18

**Authors:** Chia-Huan Chung, Liang-Ting Wu, Daniel Muara Sentosa, Chun-Chieh Ho, Po-Wei Chi, Wen-Chia Hsu, Kuo-Wei Yeh, Chung-Chieh Chang, Bing Joe Hwang, Maw-Kuen Wu, Jyh-Chiang Jiang, Chien-Chieh Hu, Yu-Cheng Chiu

**Affiliations:** † Graduate Institute of Applied Science and Technology, National Taiwan University of Science and Technology, No. 43 Keelung Road, Sec 4, Taipei 10607, Taiwan; ‡ Department of Chemical Engineering, National Taiwan University of Science and Technology, No. 43 Keelung Road, Sec 4, Taipei 10607, Taiwan; § Institute of Physics, Academia Sinica, No. 128, Section 2, Academia Road, Taipei 11529, Taiwan; ∥ Department of Mechanical Engineering, Chung Yuan Christian University, No. 200, Chungpei Road, Chungli District, Taoyuan, 32023, Taiwan; ⊥ GUS Technology, Taoyuan, 32063, Taiwan

**Keywords:** Li-metal batteries, Anode-free, Copolymer, Self-healing, Single-ion conducting, Artificial interface

## Abstract

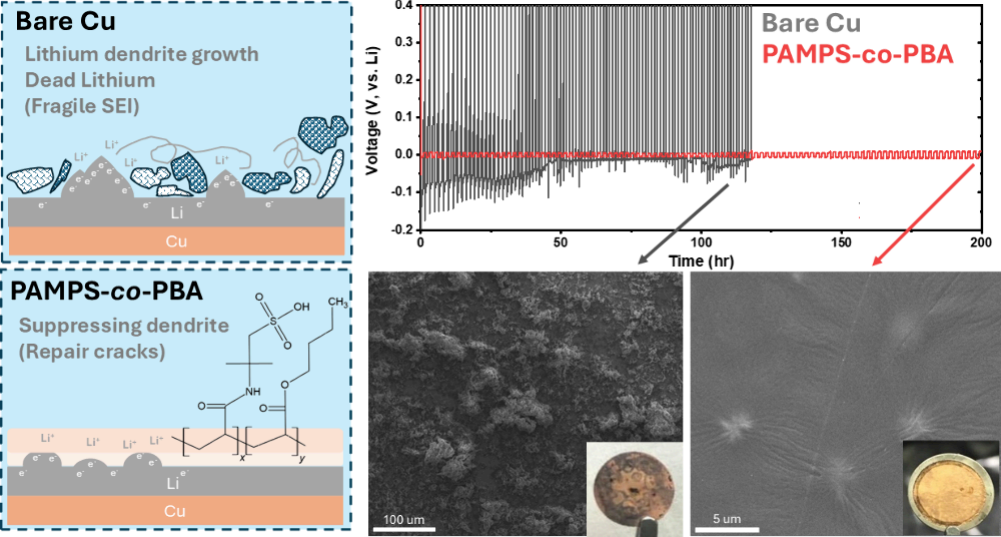

The design of anode-free batteries presents an attractive approach to the lithium metal battery. However, challenges such as uneven plating of lithium and poor Coulombic efficiency limit their commercially viable applications. In response to these challenges, this study introduces poly{(2-acrylamido-2-methylpropanesulfonic acid)-*co*-(butyl acrylate)} (PAMPS-*co*-PBA), an artificial interface engineered to enhance the cyclic stability of batteries by fortifying the solid electrolyte interphase (SEI) and enabling self-healing and single-ion conductivity. Synthesis outcomes, validated by FTIR and ^1^H NMR spectra, demonstrate successful production of PAMPS-*co*-PBA. Experimental results, including analyses of surface morphology, tensile strength, and Li plating/stripping tests, demonstrate the effectiveness of PAMPS-*co*-PBA in preventing dendrite formation and achieving >99% Coulombic efficiency. SEM analysis reveals better surface morphology and minimal lithium deposits for PAMPS-*co*-PBA compared with bare copper and other alternative interfaces. XPS analysis confirms the self-healing and single-ion conducting attributes of PAMPS-*co*-PBA postcycling. Density functional theory calculations elucidates the interface’s behavior, confirming a pathway for Li-ion movement facilitated by the sulfonic acid group. Ab initio molecular dynamics simulations highlight the potential for SEI formation, shedding light on the influence of LiTFSI on interface protection. Anode-free full cell testing demonstrates PAMPS-*co*-PBA enhancement in stability over bare copper, with 1.6 times capacity retention over 50 cycles, primarily attributed to self-healing and dendrite suppression. Nonetheless, observed capacity fading after prolonged cycling suggests the optimization of Li salt choice. Overall, PAMPS-*co*-PBA presents a promising solution for enhancing battery performance through advanced interface engineering.

## Introduction

Increasing demand for electronic devices requires an escalation in the research and production of energy storage systems, such as batteries. As more advancements have been made in this direction, pursuing lighter batteries with a higher capacity is an emerging trend. Batteries employing graphite as the anode face an inherent limitation of a relatively low energy density ranging from 250 to 350 Wh kg^–1^.^1,2^ Currently, the electric vehicle market expects an energy density greater than 500 Wh kg^–1^ to meet full-scale applications.^3^ Thus, research has focused on anode materials such as silicon, germanium, lithium, etc.^4,5^ In order to achieve the needed energy density, a lithium metal battery was considered the most attractive candidate with its low electrochemical redox potential (−3.04 V vs SHE)^6^ and ultrahigh theoretical specific capacity (3860 mAh g^–1^).^4^ To further elevate the energy density, the concept of anode-free batteries has been introduced. In contrast to traditional lithium batteries, anode-free batteries require an excess of lithium to ensure extended operation,^7^ demanding high Coulombic efficiency for sustained battery operation over an extended duration. Despite this challenge, anode-free batteries bring increased energy density (38.5%) and volumetric energy density (85.5%).^8^ This advancement aligns with consumer expectations for lighter weight and long-lasting batteries.

While anode-free batteries offer impressive energy density, there are challenges hindering their commercial use. The depletable lithium is supplied by cathode materials, such as LiFePO_4_ (LFP) or LiNiCoMnO_2_ (NCM),^7,9,10^ which are plated on a current collector during the charging process. Simultaneously, the highly reactive lithium metal reacts with the electrolyte, creating a solid electrolyte interphase (SEI) on the lithium surface. This interface is prone to cracking and rebuilding, as the lithium volume fluctuates during charge and discharge processes. When lithium ions are consumed by parasitic reactions, it leads to low Coulombic efficiency. Moreover, nonuniform lithium plating increases the risk of a short-circuit due to lithium dendrite permeation. Over the past 20 years, several strategies to maintain a long-term operation have been derived to hinder lithium metal growth, including 3D framework design,^11−13^ electrolyte optimization,^14^ and artificial interfaces.^9,10^ 3D framework designs are made to enhance volumetric fluctuation and charge distribution, effectively suppressing dendrite formation.^12,13^ However, these designs may lead to reduced energy density and excessive electrolyte consumption due to the increase in the surface area.^8^ On the other side, electrolyte optimization and an artificial interface would effectively reduce the electrolyte consumption issue. Electrolyte optimization, through compositional or concentration adjustments, aims to maintain lithium-ion concentration in the electrolyte.^14−16^ Utilizing specific lithium salts can generate inorganic SEI layer, rich in lithium-conductive derivatives, which is hypothesized to reduce nucleation overpotential and promote uniform lithium deposition.^15,17,18^ Nevertheless, high concentration electrolytes present another challenge on the reduction of ionic conductivity due to a viscosity increase.^18^ Moreover, ruptured SEI from volume fluctuation would occur due to insufficient SEI mechanical strength.

Artificial interfaces represent a key strategy that provides mechanical strength and chemical stability to counter the fluctuating volume and the high reactivity of lithium metal, respectively. The tight interface prevents the exposure of fresh lithium, repetitive SEI formation, and suppressing lithium dendrites, resulting in a significantly enhanced battery life cycle.^9^ However, the high mechanical strength of the interface does not adequately prevent the formation of lithium dendrites. Liu et al. highlighted the importance of artificial interfaces with high ionic conductivity in controlling concentration polarization and current density, which are crucial factors in postponing dendrite formation.^19^ To address the dual requirements of mechanical robustness and high ionic conductivity, copolymer-based modifications such ethylene-vinyl acetate have been investigated.^20^ The ether segments provide high ionic conductivity by a lone pair from oxygen, while ethylene segments enhance mechanical flexibility by a long chain. Utilization of various properties from different monomer variations is expected to improve mechanical flexibility while ensuring essential ionic conductivity, leading to effective dendrite suppression. However, artificial interfaces remain easily ruptured due to lithium volume fluctuations during cycling. Self-healing polymers have been regarded as a potential solution to repair any interfacial damage during volume fluctuation.^21−23^ However, current applications of copolymer interface still focus primarily on lithium metal and silicon electrodes.^21−23^

Thus, a workable artificial interface needs to be mechanically flexible to resist the permeation of lithium dendrites and has the feature of enduring volume changes. To tackle interfacial strength problems, we propose self-healing and single-ion conductivity as an artificial interface. 2-Acrylamido-2-methylpropanesulfonic acid (AMPS) has been identified as a material that exhibits both of these behaviors. However, the polymer derived from AMPS lacked softness and was prone to shattering. To address the mechanical shortcomings of AMPS, we incorporated the softness of butyl acrylate (BA). The polymer derived from BA had a soft network that cannot withstand volumetric fluctuations in lithium metal, potentially resulting in cracks on the interface during lithium plating and stripping. Their respective limitation can be overcome by employing them as a copolymer. It is possible to amend the deficiency of the AMPS mechanical property by incorporating softness of BA through copolymerization. After the synthesis and purification process, the copolymer does exhibit balanced mechanical strength and softness. In addition, its long-term cyclic stability is revealed by a plating and stripping test. During the plating and stripping tests, our copolymer showed a low overpotential and good Coulombic efficiency. After 100 cycles of lithium plating/stripping, the SEM image displayed clean and flat surface of the electrode. Furthermore, DFT calculations showed that SO_3_^–^ functional group of AMPS would interact with Li^+^, contributing to the suppression of dendrite formation. Through these results, PAMPS-*co*-PBA demonstrates the functionality to suppress dendrite growth with only small wrinkles on the surface.

## Experimental Section

### Materials

Chemicals and materials used in this work were sourced from reputable suppliers. Butyl acrylate (BA, ≥99%), 2-acrylamido-2-methylpropanesulfonic acid (AMPS, 99%), lithium bis(trifluoromethanesulfonyl)imide (LiTFSI), 1,3-dioxolane (DOL), 1,2-dimethoxyethane (DME), lithium nitrate (LiNO_3_), and methanol were procured from Sigma-Aldrich. The lithium disc and LFP electrode was obtained from UBIQ Technology Co., Ltd. Butyl acrylate underwent purification through alumina column chromatography prior to usage. 2,2-Azobis(2-methylpropionitrile) (AIBN) was purchased from UniRegion Bio-Tech. Dialysis of the copolymer was accomplished using Spectra/Por 6 Dialysis Membrane Prewetted RC Tubing with a 1 kD MWCO. Unless specified, other compounds did not undergo additional purification and were utilized as received.

### Synthesis of PAMPS-*co*-PBA Copolymer

The polymer was synthesized through radical polymerization from two types of monomers, namely, AMPS and BA. The synthesis involved two round-bottomed flasks. In the first flask, BA (4.30 mL, 0.03 mol) was combined with 30 mL of methanol. In the second flask, AMPS (4.14 g, 0.02 mol) and AIBN (0.032 mg, 0.0002 mol) were added. Prior to the mixing process, vacuuming was performed for 15, 10, and 5 min with 5 min of purging with argon after each vacuum. The molecular ratio of the monomer to initiator was maintained at 250:1. Magnetic stirring was applied for 30 min before combining the powder and liquid flasks. The solution of BA and methanol was then injected into the flask containing AMPS and AIBN, and the mixture was stirred at 1150 rpm at 65 °C for 24 h. The copolymer, PAMPS-*co*-PBA (40:60), was purified using magnetic stirring and a dialysis membrane, dispersed in methanol. After solvent evaporation, the result obtained was viscous transparent liquid of PAMPS-*co*-PBA. In addition, PAMPS and PBA were also synthesized as control group. The experimental procedure is shown in Figure S1.

### Artificial Interface Layer on Copper

The application of the copolymer PAMPS-*co*-PBA onto copper foil was achieved through spin coating, resulting in a uniformly applied, flat protective layer. Before the spin-coating process, the PAMPS-*co*-PBA solution underwent overnight drying in a vacuum system. Methanol was subsequently added as a solvent, and the mass concentration of PAMPS-*co*-PBA in the solution was adjusted to 14.9 wt %. Once the solution was prepared, it was carefully dropped onto the copper foil, and the spin coater was operated at 4000 rpm. Subsequently, the foil was transferred to an oven and dried at 80 °C for a duration of 3.5 h.

### Characterization

Attenuated total reflectance-Fourier-transform infrared spectroscopy (ATR-FTIR) was used to identify the functional group of polymers, in which wavelengths were recorded from 800 to 4000 cm^–1^. NMR graphics were obtained from nuclear magnetic resonance spectroscopy (Bruker Avance III HD-600). In the ^1^H NMR analysis, the product was dissolved in DMSO-*d*_6_ (δ = 2.51 ppm). MeOH signal at δ = 3.17 ppm represents the solvent residue followed by the HDO peak at 4.43 ppm. Another ^1^H NMR δ (ppm): 0.88–0.92 (t, 3H, −OCH_2_CH_2_CH_2_CH_3_), 1.26–1.60 (−OCH_2_CH_2_CH_2_CH_3_; −NHC[CH_3_]_2_CH_2_S−), 2.89 (s, 2H, −NHC[CH_3_]_2_CH_2_S−), 3.57 (t, 2H, −OCH_2_CH_2_CH_2_CH_3_), 7.63 (s, 1H, −NHC[CH_3_]_2_CH_2_S−). PAMPS-*co*-PBA-modified copper was further assembled into a coin cell (Li∥PP∥PAMPS-*co*-PBA). The artificial interface electrode was characterized by X-ray photoelectron spectroscopy (XPS, PHI 5000 VersaProbe III) after one lithium plating/stripping cycle at 0.5 mA cm^–2^ and 0.5 mAh cm^–2^. Before XPS analysis, the disassembled electrode was rinsed with a 1:1 (v/v) DME/DOL solution to remove residual electrolytes. The Li 1s spectrum was acquired by using five scans to achieve a satisfactory signal-to-noise ratio. To investigate long-term cycling effects, electrodes cycled 30 times were disassembled and cleaved. One half was used to characterize the PAMPS-*co*-PBA coating after 30th cycles, and the other half was rinsed with methanol to remove the coating and expose the underlying copper surface for analysis. The electrode surface morphology and thickness were checked by a field emission scanning electron microscope (FE-SEM, HITACHI S4200) and an energy-dispersive spectrometer (EDS, OXFORD MAX^N^ 50).

### Mechanical Tensile Test

A tensile test was conducted to observe the mechanical property of polymer by using universal testing instruments (Shimadzu EZ-EX). PAMPS-*co*-PBA solution was cast into a Teflon mold and left to rest for 3 days to eliminate air bubbles inside the sample. Subsequently, the sample was dried by heating in the oven for 24 h at 80 °C. After the drying process, the sample was left to rest for another 12 h. Finally, a transparent and clear gel was obtained after sample removal from mold. To prepare the sample to be subjected for the self-healing test, prepared film was cut in half using a razor blade, then two separated ends were attached together. The sample was left for 24 h, which results in a self-healed copolymer film. After the self-healing process is complete, pristine and self-healed samples were then subjected to a tensile test.

### Electrochemical Measurements

Electrochemical testing was conducted using CR2032-type coin cells assembled with a 15.6 mm diameter lithium foil and a 14 mm current collector. The electrolyte consisted of 1 M LiTFSI in DME/DOL (1/1, v/v), supplemented with 2 wt % LiNO_3_ as an additive. Our artificial interface was assembled on copper as the working electrode, along with lithium foil and a separator, for lithium plating and stripping tests. A constant current density of 0.5 mA cm^–2^ was applied, and the voltage range was limited to 3.0 V to −3.0 V. After 100 cycles, the coin cell was disassembled. Subsequently, the morphology of the PAMPS-*co*-PBA electrode sample was identified via scanning electron microscopy (SEM). Electrochemical impedance spectroscopy (EIS) for a CR2032 coin cell was performed on an Autolab PGSTAT302N electrochemical workstation using an AC amplitude sweep from 0.1 MHz to 0.1 Hz. Distribution of relaxation times (DRT) was performed on EISART software, which was developed by Hangyue et al.^24^ For the cycle life test, the PAMPS-*co*-PBA electrode/separator/LiFePO_4_ coin cell, with 0.8 mL of electrolyte, was subjected to testing. The voltage range was set between 3.0 and 4.0 V, with the C-rate maintained at 0.1C (theoretical capacity of LiFePO_4_ is 140 mAh g^–1^).

### Density Functional Theory (DFT) Calculation

DFT calculations were conducted using the Vienna ab initio Simulation Package (VASP) along with the projector augmented wave (PAW) method. We employed the Perdew–Burke–Ernzerhof (PBE) generalized gradient approximation (GGA) functional to describe exchange and correlation interactions. The plane wave cutoff energy, electronic energy convergence, and force convergence were set to 550 eV, 10^–4^ eV, and 0.01 eV/Å, respectively. We incorporated the van der Waals correction using the DFT-D3 method and utilized a gamma point sampling for the Brillouin zone in the polymer–salt bulk. For the polymer–salt|Li/Cu interface, a 2 × 2 × 1 k-point mesh was employed. The Li/Cu surface was constructed with two layers of a 5 × 5 Li (100) surface and six layers of a 5 × 5 Cu (100) surface, and the bottom two layers of Cu were replaced with He atoms to prevent neighboring box interaction. During the simulations, the bottom five layers, including two layers of He and three layers of Cu, were kept fixed.

### Ab Initio Molecular Dynamics Simulation

The canonical ensemble (NVT) with a time step of 1 fs was employed in the AIMD simulations for this work. The temperature was set to 450 K to accelerate the chemical reactions within the simulation times. The total simulation time was 20 ps for the polymer–salt system and 8 ps for the polymer–salt|Li/Cu interface. All initial configurations were pre-equilibrated using classical molecular dynamics simulations in the Forcite module with the COMPASS II force field within the Materials Studio software. In the pre-equilibrium simulation, the temperature was set to 450 K, and the total simulation time was 10 ps.

### Atomic Charge Distribution

The atomic charge distribution was calculated by using Bader charge calculations. To obtain a normal distribution, ten configurations were selected from the last 500 fs of the simulation for both the polymer–salt and the polymer–salt|Li/Cu interface systems. The charge data were collected and represented by a Gaussian function.

## Results and Discussion

**Figure 1:**
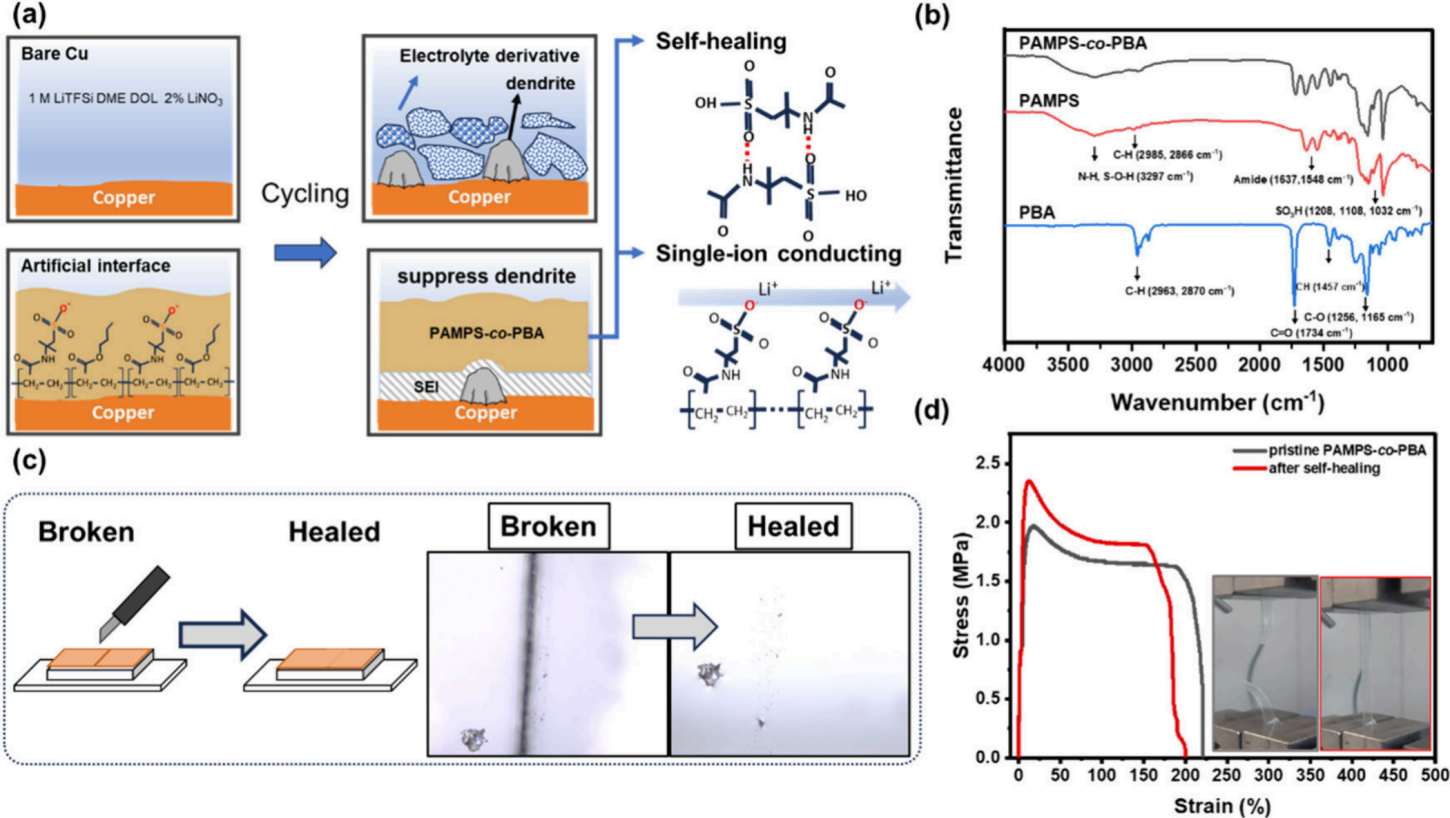
(a) Schematic illustration depicting the Li deposition behaviors on bare copper and PAMPS-*co*-PBA electrodes. (b) FTIR spectra of PBA, PAMPS, and PAMPS-*co*-PBA. (c) Optical images demonstrating the self-healing behaviors of PAMPS-*co*-PBA. (d) Tensile strength test results for PAMPS-*co*-PBA before and after self-healing.

Figure 1a illustrates a schematic diagram of PAMPS-*co*-PBA as an artificial interface designed to protect the fragile SEI and enhance cyclic stability through self-healing and single-ion conductivity. In contrast, the surface of bare copper generated several byproducts, such as LiF, LiCO_3_, and other derivatives, from the electrolyte. In our copolymer, self-healing is facilitated by the hydrogen bond from AMPS, countering volume changes in lithium, while the sulfonic acid functionality rectifies the lithium-ion transfer pathway, inhibiting lithium dendrite growth. The FTIR (Figure 1b) and ^1^H NMR (Figure S2) spectra provide the synthesis results. In the FTIR test, the results of PAMPS-*co*-PBA display signals of CH (2963, 2870 cm^–1^), C=O (1734 cm^–1^), and C–O (1256, 1165 cm^–1^), which belong to PBA;^25^ and sulfonic acid (1208, 1108, 1032 cm^–1^), amide II (1637, 1548 cm^–1^), and N–H and S–O–H groups (broad peak, 3297 cm^–1^) from PAMPS.^26−28^ In the ^1^H NMR technique, signals at δ = 0.88–0.92 ppm belong to CH_3_ in −CH_2_CH_2_CH_3_ corresponding to PBA, followed by CH_2_ peaks at 1.26–1.60 ppm; 3.57 ppm represents the CH_2_ peak of PBA in −COOCH_2_–; and signals at δ = 1.4, 2.89, and 7.63 ppm belong to hydrogen in the PAMPS group (CH_3_, CH_2_, NH). The AMPS:BA ratio calculated from NMR spectra closely matches the theoretical molecular ratio. In summary, random block PAMPS-*co*-PBA was successfully synthesized with an AMPS:BA molecular ratio of 4:6.

Figure 1c includes a schematic diagram of the cutting process and a photograph of PAMPS-*co*-PBA to prove its self-healing ability through an optical microscope (OM). In this test, the copolymer was spin-coated on top of polyvinylidene fluoride (PVDF) and left to dry. The self-healing ability of the copolymer was evident 24 h after the cut that was kept at room temperature and ambient pressure, as shown in the photograph. In addition, tensile strength measurements were done to compare the mechanical strength between pristine and self-healed copolymer films. Figure 1d shows similar elastic regimes (10%) for both pristine and self-healing samples, with yield strengths of 1.94 and 2.34 MPa, respectively. However, the self-healing sample exhibits a shorter strain length, suggesting easier fracture.

**Figure 2:**
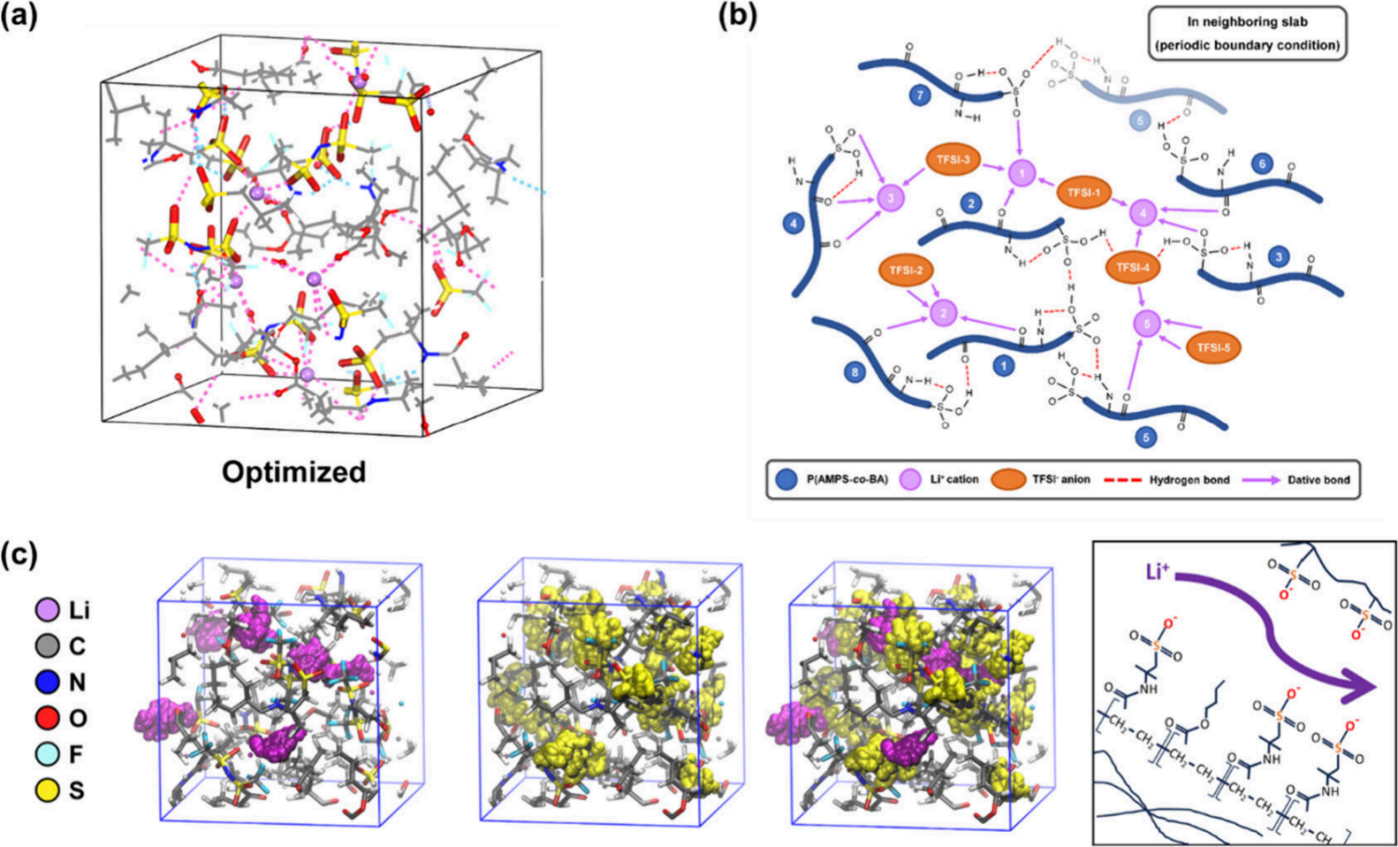
(a) Structural optimization of PAMPS-*co*-PBA and the LiTFSI salt mixture. (b) Schematic diagram illustrating the intramolecular and intermolecular interactions of a copolymer and Li salt in the battery. (c) Trajectory of atom motion for PAMPS-*co*-PBA during a 20 ps duration.

To obtain a more in-depth understanding of the self-healing capability of PAMPS-*co*-PBA, density functional theory (DFT) calculations and ab initio molecular dynamics (AIMD) simulations were used to further explore the intramolecular and intermolecular interactions of the copolymer and Li salt in the battery. An oligomer containing one AMPS molecule and one BA molecule was used to represent the copolymer of PAMPS-*co*-PBA in the simulation. Eight oligomers and five LiTFSI salt molecules were assigned to the simulation box, as depicted in Figure 2a. The simulation time intervals ranged from 0 to 20 ps, showing the status of the movement of the material within that period. The schematic diagram in Figure 2b helps to comprehend the simulation results after 20 ps. The intramolecular and intermolecular interactions between polymers and salts were counted and are summarized in Table 1. Overall, five interchain hydrogen bonds, seven intrachain hydrogen bonds, and two polymer–salt hydrogen bonds were found. Meanwhile, the dative interactions between Li^+^ and oxygen from TFSI anions and from polymers also play a crucial role, with 20 Li^+^–O interactions observed in total. The results reveal intermolecular hydrogen bonds between the sulfo group and the carboxamide group, attributed to a self-healing feature. Additionally, Figure 2c displays the trajectory of atom motion during a 20 ps simulation, revealing the pathway of lithium (purple ball) correlating with the −SO_3_H/–SO_2_– rather than the C=O group. This result suggests that the sulfo group is capable of promoting the Li-ion movement in PAMPS-*co*-PBA.

**Table 1:** Summarized Interactions in a PAMPS-*co*-PBA Self-Healing Polymer with LiTFSI Salt^a^

interactions		No. of interactions
hydrogen bond	interchain	5
	intrachain	7
	polymer–TFSI	2
dative bond	Li^+^–O_TFSI_	10
	Li^+^–O_polymer_	10

a The threshold of hydrogen and dative bond length was set to 2.45 Å.

**Figure 3:**
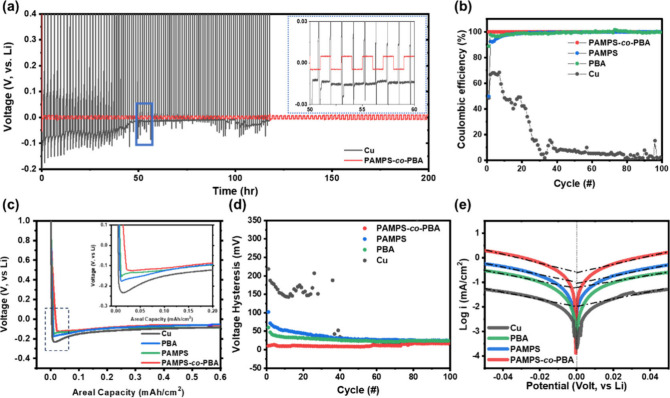
Electrochemical performance comparison of Li/Cu half cells without/with a modified coating. (a) Cycle stability comparison of bare Cu and PAMPS-*co*-PBA, both at a deposition capacity and current density of 0.5 mA cm^–2^ and 0.5 mAh cm^–2^, respectively. (b) Coulombic efficiency of the Li plating/stripping process on bare Cu and modified Cu. (c) Voltage–capacity curve of bare Cu and modified Cu during the first cycle discharge process. (d) Voltage hysteresis of bare Cu and modified Cu at different cycles. (e) Tafel plot of bare Cu and modified Cu half cells.

The artificial interface was coated onto a copper foil with the detailed method presented in Figure S3a. The thickness of the artificial interface was about 1.2 μm, as shown in the EDS line scan (Figure S3b and S3c). We used these electrodes for lithium plating–stripping measurements, utilizing lithium metal as both counter and reference electrodes to assess the performance of lithium deposits during the charge and discharge processes, and the results are depicted in Figure 3a. The signal from the PAMPS-*co*-PBA electrode notably differed from that of the bare copper electrode. As discussed by Huang et al., the results revealed voltage fluctuation with lithium.^29^ The voltage of the bare copper electrode exhibited an elevation at the end of the stripping process, suggesting irreversible lithium consumption due to continuous SEI formation and the generation of a dead lithium layer. The calculated Coulombic efficiency from plating and stripping, as displayed in Figure 3b, shows that the bare copper electrode has an initial cycle efficiency of ∼40%, indicating significant utilization of a lithium ion for SEI formation. Subsequently, a rapid decay was observed, likely attributed to fresh lithium repeatedly coming into contact with the electrolyte, causing fragile SEI and fluctuations in the lithium volume. In contrast, the polymer interfaces of PBA, PAMPS, and PAMPS-*co*-PBA exhibited much higher Coulombic efficiency, achieving >99% after 20 cycles. This suggests that these interfaces prevent continuous reaction with the electrolyte during lithium metal generation. However, the Coulombic efficiency of PBA and PAMPS samples does not reach >99% in the initial 20 cycles, as further detailed in Figure S4a and S4b. The voltage lift at the end of stripping indicates the formation of a dead lithium layer during the cyclic process. More on this topic will be discussed, along with SEM analysis.

Nucleation overpotential, defined as the difference between the minimum potential during initial nucleation and the subsequent plateau potential during growth, was analyzed to assess the lithium affinity of the interface (Figure 3c, Table S1). The nucleation overpotentials were shown as −58 mV for PAMPS-*co*-PBA, −74 mV for PAMPS, −103 mV for PBA, and −132 mV for bare copper, respectively. As the nucleation overpotential is closely related to the free energy barrier of lithium deposition that is influenced by both SEI thickness and lithium affinity, we further investigated these factors using voltage hysteresis and Tafel plots in Figure 3d,e. Figure 3d illustrates the voltage hysteresis, a proxy for the SEI thickness. Bare copper exhibited the highest hysteresis (approximately 150 mV), indicating a thicker SEI layer. Conversely, PAMPS-*co*-PBA showed the lowest hysteresis (10.3 mV), while PBA and PAMPS displayed intermediate values (44.4 and 32.2 mV, respectively). To evaluate lithium-ion transport kinetics, we employed Tafel plots (Figure 3e) to determine the exchange current density, which is a measure of reaction rate. PAMPS-*co*-PBA exhibited the highest exchange current density of 0.342 mA cm^–2^, followed by PAMPS (0.126 mA cm^–2^), PBA (0.075 mA cm^–2^), and bare copper (0.016 mA cm^–2^). The enhanced exchange current densities observed for PAMPS-*co*-PBA and PAMPS can be attributed to their single-ion conducting characteristics, which facilitate lithium-ion transfer at the copper interface. This also supports our simulation results (Figure 2c), demonstrating that the sulfo group promotes lithium-ion transport.

To further investigate the interfacial resistance, we conducted an EIS analysis before and after cycling (Figure S5a). Prior to cycling, all coatings exhibited large semicircles indicative of high interfacial resistance. This result occurs due to foreign surface films formed during cell assembly, as reported by Hobold et al.^16^ After cycling, the EIS spectra became more complex due to the formation of the Cu/Li/SEI/artificial interface. To quantify the effective resistance (*R*_eff_), we performed distribution of relaxation times (DRT) analysis in the midfrequency region (10^4^ to 10^1^ Hz; Figure S5b), and the calculated *R*_eff_ values are presented in Figure S5c. Notably, the *R*_eff_ values exhibited an inverse relationship with the exchange current density, with PAMPS-*co*-PBA demonstrating the lowest *R*_eff_, followed by PAMPS, indicating superior ionic conductivity. This trend corroborates the results presented in Figure 3e. Conversely, PBA exhibited a lower exchange current density and a higher *R*_eff_ due to the lack of ion transfer promotion feature. Moreover, when comparing the DRT peak frequencies in the midfrequency range across all samples, the PAMPS-*co*-PBA sample exhibits a relatively higher characteristic peak frequency. This indicates a shorter transition time from transient to steady-state electrochemical reactions, suggesting improved ionic conductivity.

The superior performance of PAMPS-*co*-PBA can be attributed to its copolymer structure, which leverages the distinct functionalities of its constituent monomers. Similar to how ethylene-vinyl acetate copolymers utilize ether segments for high ionic conductivity and ethylene segments for enhanced mechanical flexibility, PAMPS-*co*-PBA combines the ionic conductivity and self-healing of PAMPS with the flexibility properties of PBA. This synergistic effect allows for optimized lithium-ion transport and robust interface stability, ultimately leading to an improved electrochemical performance. Consistent with Liu et al.’s report that mentioned artificial SEI with high ionic conductivity accelerates lithium-ion transport and reducing concentration polarization as well as dendrite growth,^19^ plating and stripping tests shown in Figure 3b revealed that PAMPS-*co*-PBA exhibited the best cycle life and Coulombic efficiency. The inherent self-healing ability of these polymers is proven to protect the internal SEI, preventing repeated electrolyte exposure to lithium, contributing to high Coulombic efficiency and low voltage hysteresis. In contrast, bare copper and PBA-coated electrodes failed to effectively optimize the interface and exhibited poor performance. The substantial volume changes during cycling may lead to SEI rupture and regeneration, depleting lithium ions and reducing cycle life. While PAMPS shares similar functional groups with PAMPS-*co*-PBA, its brittleness leads to cracking during cycling. This results in higher voltage hysteresis and a higher overpotential in the first cycle voltage-capacity curve, suggesting a potential SEI formation within the cracks.

**Figure 4:**
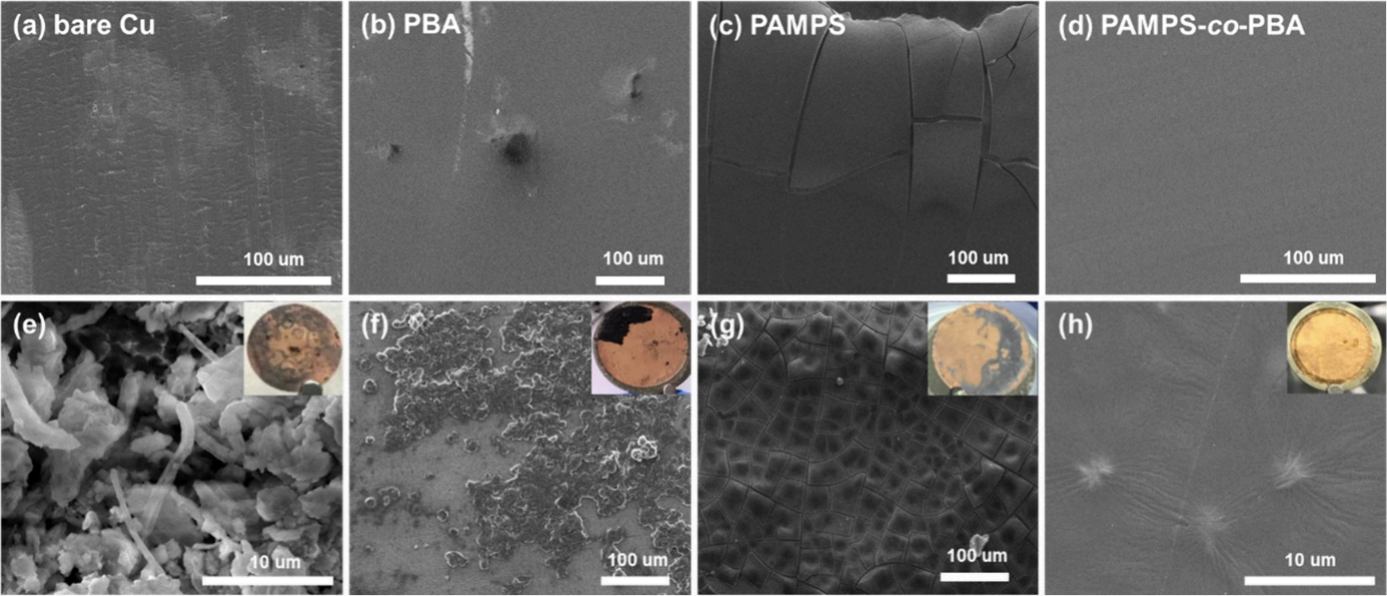
SEM morphology of lithium deposition before cycling on different electrodes: (a) bare Cu, (b) PBA, (c) PAMPS, and (d) PAMPS-*co*-PBA. Optical images (insets) show bare and modified copper electrodes, along with corresponding SEM images after 100 cycles for each type of electrode: (e) bare Cu, (f) PBA, (g) PAMPS, and (h) PAMPS-*co*-PBA. The experiments were conducted in an electrolyte comprising 1 M LiTFSI, DME/DOL (1/1, v/v), and 2 wt % LiNO_3_, with lithium plating/stripping current density of 0.5 mA cm^–2^.

To comprehend the alterations in surface morphology, SEM analysis was conducted on all samples before and after Li plating/stripping cycles, as shown in Figure 4. Notably, the surface of bare copper appears smooth, as in Figure 4a. However, a messy and gray appearance was present after lithium deposition observation postcycling, indicative of dead lithium formation (Figure 4e). The SEM image further reveals filamentary and foliated lithium metal on the copper surface, representing dead lithium that contributes to Coulombic efficiency loss.^30^ For the PBA electrode, both the digital photograph and SEM image (Figure 4b and f) reveal deposits on the surface, a finding further confirmed by EDS mapping (Figure S6). Significantly, the deposits correlate with the sulfur signal, and LiTFSI was identified as the sole source of sulfur in this cell. Moreover, the artificial interface of PBA is conceived to be too soft and unable to withstand lithium permeation, resulting in the formation of an SEI on the surface. On the other hand, the artificial interface of PAMPS reveals a surface full of brittle cracks, both in pristine and after 100 cycles (Figure 4c and g). These cracks originate from the lack of softness of the material. Consequently, the low Coulombic efficiency and high voltage hysteresis of PBA and PAMPS may arise from mechanical property defects. In contrast, PAMPS-*co*-PBA coated electrodes of both precycling and postcycling show relatively flat surface, as shown in Figure 4d and h. Additionally, wrinkles on the surface suggest that lithium dendrite growth is impeded by the flexible, self-healing interface. Therefore, those properties play a crucial role in withstanding volume fluctuations during the cycling process.

**Figure 5:**
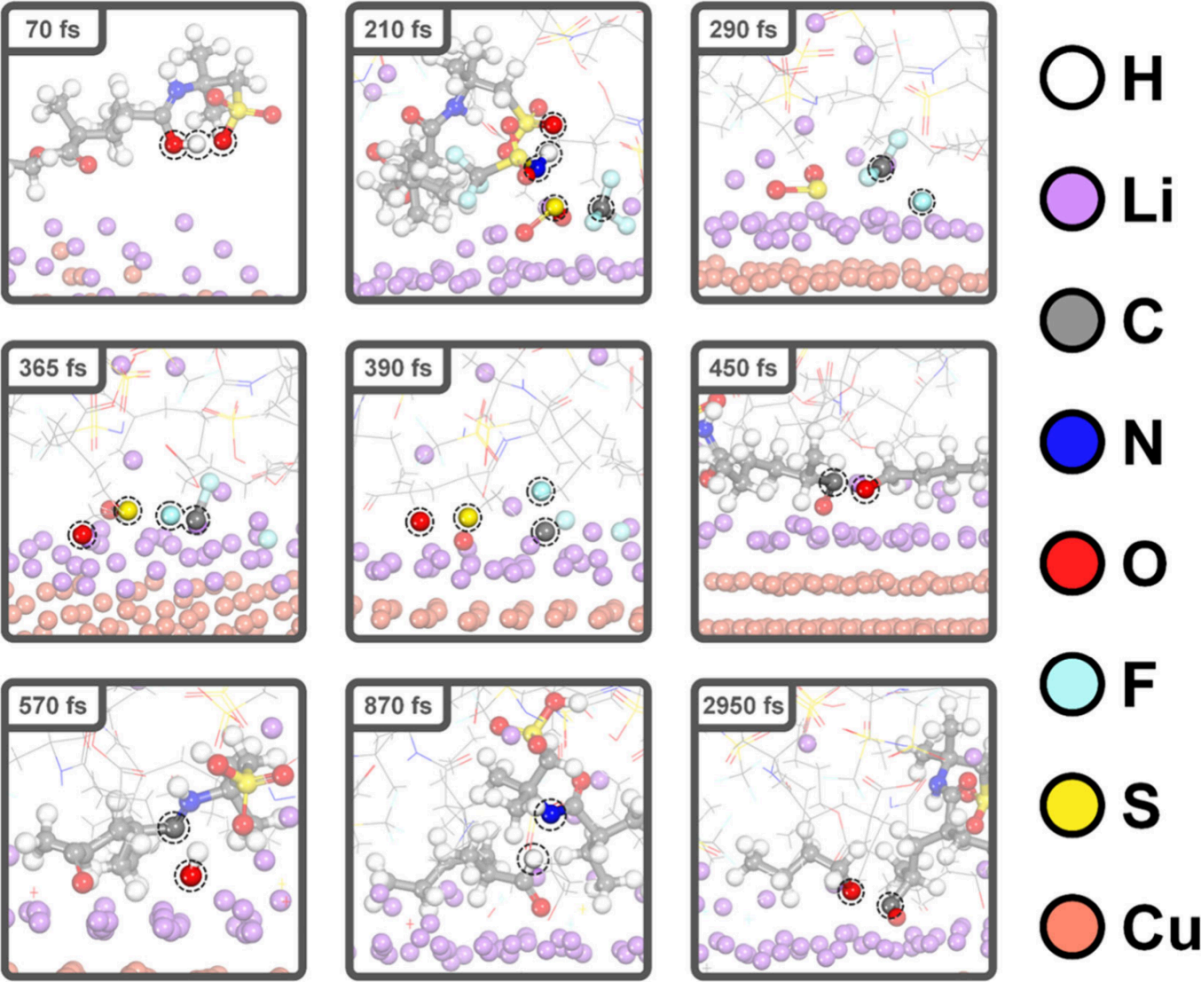
Snapshots of the reaction of PAMPS-*co*-PBA and the LiTFSI unit at the lithium metal interface.

To investigate the interfacial phenomenon between the PAMPS-*co*-PBA protecting layer and the anode surface, an AIMD simulation was performed. We formulated two systems by considering different environments: one with Li salt in proximity to the lithium metal and another with Li salt positioned far away. The initial state of the system is shown in Figure S7. The electrode simulation involved two layers of lithium (100) and four layers of copper, mimicking the structure of the PAMPS-*co*-PBA electrode with a focus on lithium plating occurring on the copper surface. Figure 5 and Figure S8 show snapshots of representative decomposition of PAMPS-*co*-PBA and LiTFSI during an 8 ps AIMD simulation when Li salt was positioned in proximity to and far away the anode surface, respectively. The summarized results can be found in Table 2 and Table S2. During the simulation, the reactant was represented using a ball-and-stick model, while the unreacted material was shown as a wireframe. In Figure 5, the anion of TFSI undergoes decomposition adjacent to the lithium slab in a step-by-step process over 210 and 390 fs. Specifically, the nitrogen of TFSI was protonated by the sulfonic acid of the AMPS segment, initiating a sulfur–nitrogen (S–N) bond and a sulfur–carbon (S–C) bond breaking mechanism around ∼210 fs, resulting in the formation of SO_2_ and CF_3_ species. Following this, the two S–O and three C–F bonds of the anion fragment broke at time intervals ranging from 290 to 390 fs, leading to the formation of Li_2_S, Li_2_O, and LiF inorganic compounds near the lithium surface. This trend aligns with observations from related works, where the anion continually broke and formed LiF with Li_2_S on the lithium surface.^31^ In the case of PAMPS-*co*-PBA, the C=O of AMPS underwent protonation by sulfonic acid at 70 fs, followed by C–(OH) cleavage at 570 fs. Furthermore, these snapshots illustrated that the COOR group of BA became fragmented via the C_carbonyl_–O_ethereal_ bond breaking at 450 and 2950 fs, accompanied by the formation of lithium alkoxides (ROLi). At 870 fs, the resulting unsaturated alkoxide (RCO) deprotonates the amide group, forming RCHO and the deprotonated amide. Moreover, Figure S8 reveals analogous simulation outcomes, showing COOR bond cleavage adjacent to the lithium metal at 430 and 2000 fs. Both occurrences imply the cleavage of the COOR group in PAMPS-*co*-PBA, contributing to the formation of SEI on the lithium surface. Additionally, another notable event was the cleavage of TFSI; the nitrogen of TFSI was protonated by the sulfo group at 130 fs, resulting in the formation of cleavage fragments HNSO_2_CF_3_ and SO_2_CF_3_ at 150 fs via S–N bond breaking. This phenomenon aligns with studies reported in previous related works.^31,32^ This finding is also evident in Figure S8 and consistent with the observations in Figure 5. The partial cleavage of TFSI, induced by nitrogen protonation, accelerates TFSI decomposition and forms diverse SEI components. Notably, the ester group decomposition on the Li/Cu anode surface agrees with our previous computational work, which indicated that the ester group would be activated on the Li/Cu surface by the presence of the Cu current collector.^33^ On the other hand, the formation of inorganic species such as LiF, Li_2_O, and Li_2_S, is notable for its documented ability to suppress dendrite growth.^34^ The stiffness of these inorganic species is anticipated to contribute to suppressing dendrite growth on the lithium metal side.

**Table 2:** Catalog of PAMPS-*co*-PBA and LiTFSI Decomposition Reactions and Approximate Occurring Time During AIMD Simulations when LiTFSI Is near the Lithium Surface

species	time (fs)	reaction
PAMPS-*co*-PBA	70	R-SO_3_H + R′C(=O)NHR′′ → R-SO_3_ + R′C(OH)NHR′′
PAMPS-*co*-PBA, TFSI	210	N(SO_2_CF_3_)_2_ + R-SO_3_H → HNSO_2_CF_3_ + SO_2_ + CF_3_ + R-SO_3_
TFSI	290	CF_3_ → CF_2_ + F
TFSI	365	SO_2_ → SO + S, CF_2_ → CF + F
TFSI	390	SO → S + O, CF → C + F
PAMPS-*co*-PBA	450	R(C=O)OR′ → R(C=O) + R′-O
PAMPS-*co*-PBA	570	R′C(OH)NHR′′ → R′CNHR′′ + OH
PAMPS-*co*-PBA	870	R′C(=O)NHR′′ + R(C=O) → R’C(=O)NR′′ + RCHO
PAMPS-*co*-PBA	2950	R(C=O)OR′ → R(C=O) + R′-O

**Figure 6:**
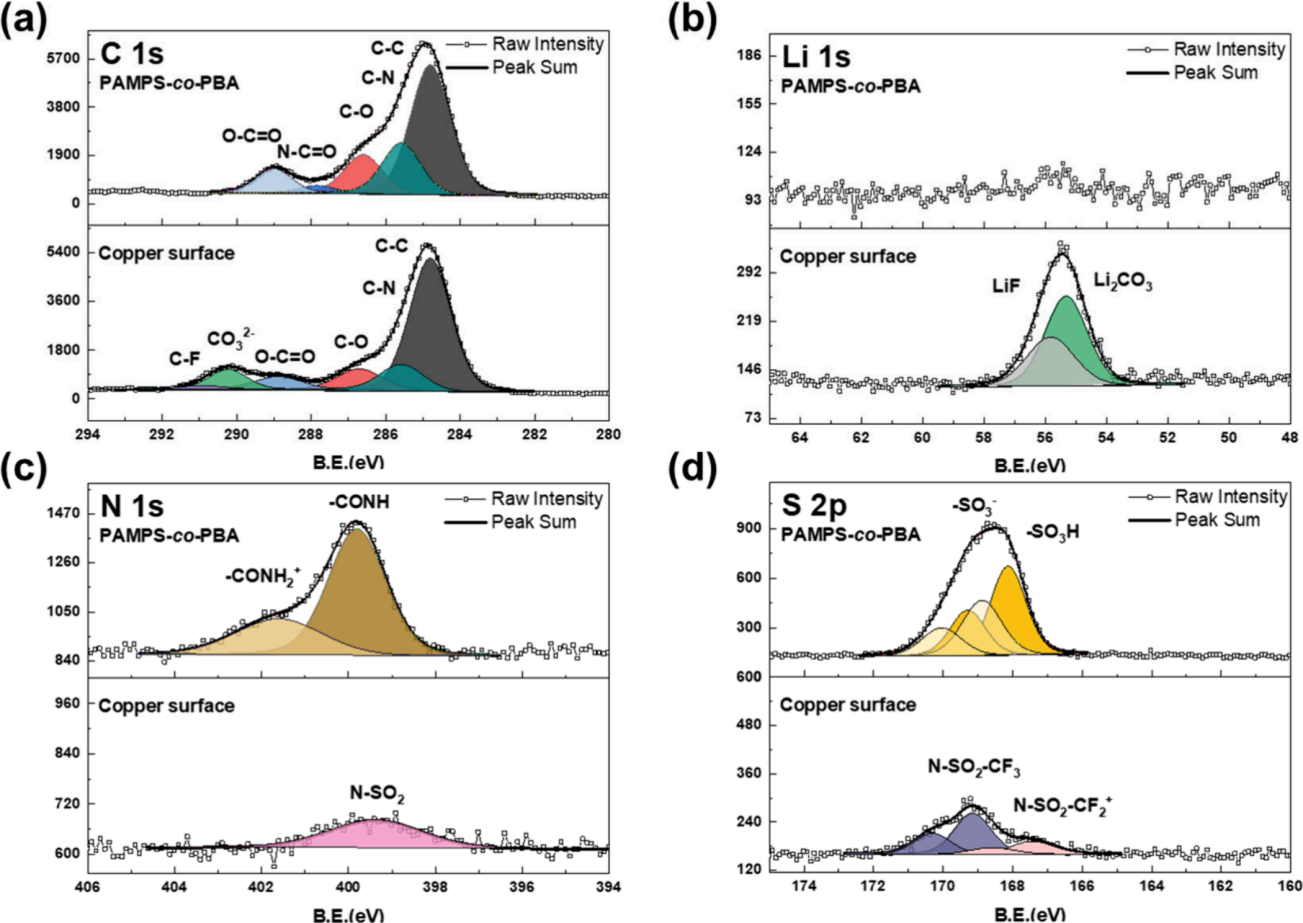
XPS spectra of (a) C 1s, (b) Li 1s, (c) N 1s, and (d) S 2p for the bare copper surface and the PAMPS-*co*-PBA-modified copper surface after one cycle of Li plating and stripping processes.

XPS analysis was conducted to examine the composition of the interface on both bare Cu and PAMPS-*co*-PBA surfaces after Li plating and stripping processes. The C 1s spectrum of PAMPS-*co*-PBA (Figure 6a) deconvolutes into five distinct areas with binding energies of 284.8, 285.6, 286.6, 287.8, and 289.0 eV assigned to C–C, C–N, C–O, N–C=O, and O–C=O, respectively. These peaks represent components originating from PAMPS-*co*-PBA without any additional peaks. On the other hand, the analysis of bare Cu after cycling displayed peaks for C–C, C–N, C–O, O–C=O, and CO_3_^2–^ in sequence. These peaks are likely attributed to electrolyte derivatives. Additionally, the C 1s spectrum exhibits a peak at around 290.9 eV on the bare Cu surface, which belongs to the decomposition of NSO_2_CF_2_ from LiTFSI.^31^ The Li 1s spectrum was employed to identify the deposits after reaction (Figure 6b). The signal on bare Cu consists of peaks of Li_2_CO_3_ (55.3 eV) and LiF (55.8 eV). Furthermore, the F 1s spectra (Figure S9) confirm the binding energy of LiF and C–F at 685.0 and 688.9 eV, respectively. Moreover, the deconvolution of N 1s and S 2p (Figure 6c and d) in the bare Cu spectra reveals signals of lithium salt decomposition. The weak N 1s signal is attributed to N-SO_2_, which is likely to be a LiTFSI derivative. The S 2p results also support this assumption, showing signals of NSO_2_ (167.4 eV) and N-SO_2_CF_3_ (169.2 eV). However, the XPS of PAMPS-*co*-PBA showed a different curve in the N 1s and S 2p spectra, indicating single-ion conductivity and self-healing properties. In the S 2p spectrum, peaks were assigned as SO_3_H and SO^_3_–^ at 168.1 and 168.9 eV, respectively.^35,36^ The SO_3_H represents sulfonic acid of PAMPS-*co*-PBA, and the signal of sulfonates (SO^_3_–^) originates from the ionization of sulfonic acid. Furthermore, the N 1s spectrum shows −CONH and −CONH^_2_+^ at 399.8 and 401.6 eV, respectively.^37,38^ According to the S 2p result, the ammonium group (−CONH^_2_+^) originates from the ionization of sulfonic acid. In summary, the sulfonates group in the PAMPS region is revealed to be a single-ion conductor, one of the key features for ion conducting ability. While PAMPS-*co*-PBA retains SO_3_H, it can be utilized for self-healing in the event of cracking. The XPS results reveal that PAMPS-*co*-PBA maintains both self-healing and single-ion conductivity features even after cycling. This explains the excellent performance of PAMPS-*co*-PBA in the Li plating and stripping test. Additional details, including binding energy, the assumed origin of signals, and attributed literature, are presented in Tables S3 and S4.

**Figure 7:**
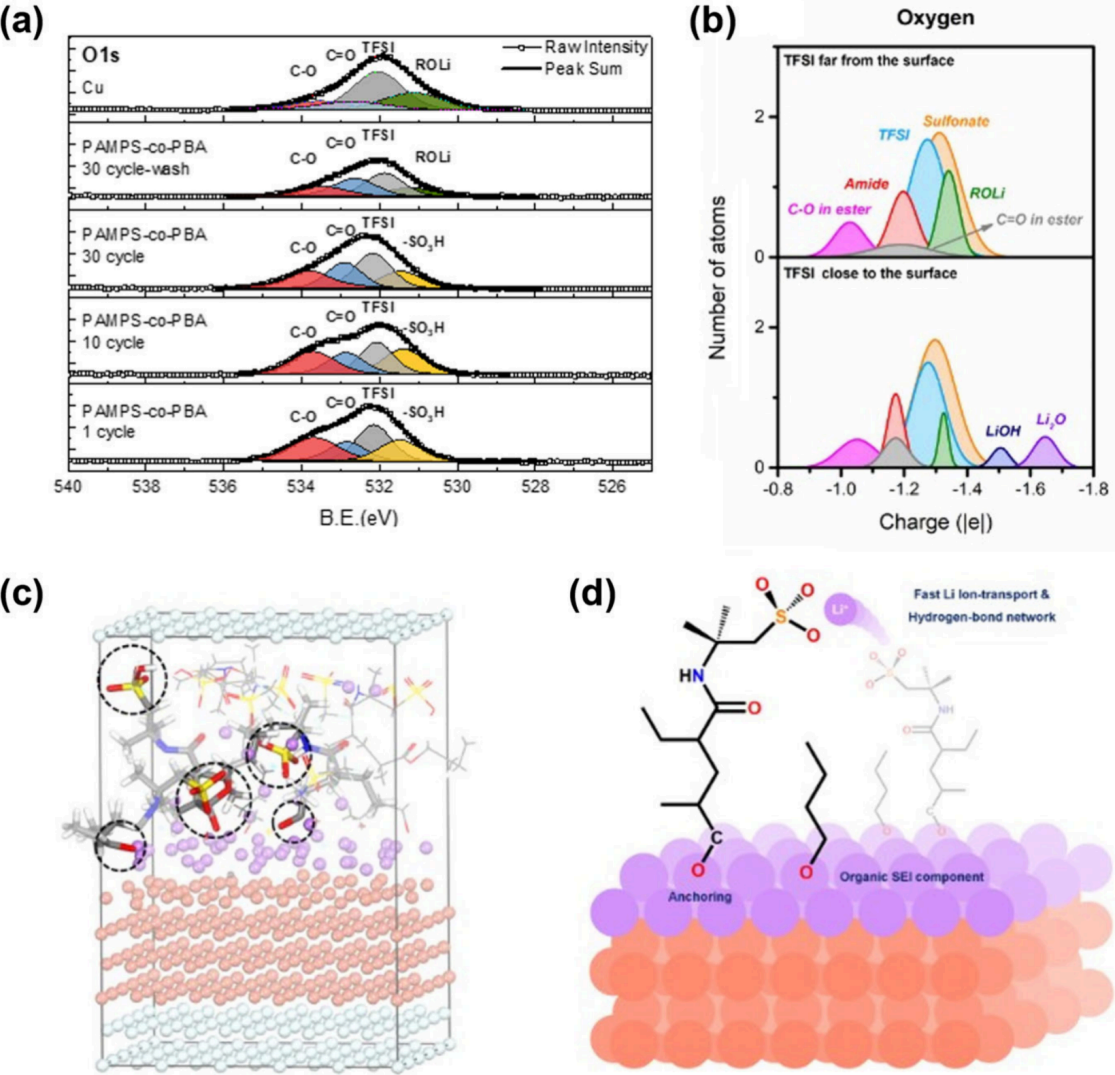
(a) XPS spectra of O 1s for the bare copper after one cycle of Li plating and stripping, and the PAMPS-*co*-PBA interface after 1, 10, and 30 cycles of Li plating and stripping. (b) Atomic charge distribution of oxygen atoms at the interface. (c) The structure of the final frame after AIMD simulation. (d) The schematic illustration of the role of PAMPS-*co*-PBA at the electrode.

To better understand the role of PAMPS-*co*-PBA in facilitating Li plating and stripping on the Cu surface, we combined the XPS spectrum of the O 1s (Figure 7a) and the oxygen atomic charge distribution (Figure 7b) from the AIMD simulation. The PAMPS-*co*-PBA surface exhibits remarkable electrochemical stability, as evidenced by its consistent performance during plating and striping. To gain more insight into the interfacial interactions between lithium and the PAMPS-*co*-PBA layer, a washing process with methanol was employed to remove the protective layer. XPS analysis of the PAMPS-*co*-PBA surface after 30 cycles and washing revealed four distinct peaks in the O 1s spectrum. These peaks present at binding energies of 533.8, 532.8, 532.0, and 531.2 eV can be attributed to C–O, C=O, N–SO_2_, and ROLi species, respectively. To further clarify the mechanism by which the copolymer interface protects the SEI through self-healing, as depicted in Figure S10, we performed XPS analysis on the cycled PAMPS-*co*-PBA coating and the exposed underlying copper surface. The detailed deconvolution of the XPS spectra is listed in Tables S5 and S6. The results suggest the formation of SEI during cycling. The N 1s and S 2p spectra demonstrated that the functional groups remained undecomposed after 30 cycles, confirming the sustained self-healing capability of the artificial interface. This self-healing effect effectively avoids SEI rupture and accommodates lithium volume changes during cycling. Consequently, inorganic derivatives were only observed in the inner layer by XPS. On the other hand, the atomic charge distribution shows high potential in predicting SEI components.^32,39,40^ For the oxygen element, the O_ethereal_ of the ester group is at ∼−1.03|e|, the O_carbonyl_ of the ester group and the amide peak are at ∼−1.18|e|, and the TFSI and sulfonate peaks are very close at ∼−1.30|e| due to their similar chemical environment. Regarding the decomposed products, the lithium alkoxide peak is at ∼−1.33|e|, providing evidence for ester decomposition. Moreover, the presence of lithium hydroxide (LiOH) and lithium oxide (Li_2_O) peaks at ∼−1.5 and ∼−1.65|e|, respectively, indicates the existence of common inorganic compounds in the SEI layer when the TFSI anion decomposes on the anode surface. Except for the LiOH and Li_2_O, all the remaining peaks have been found in the O 1s XPS spectrum, indicating that our simulation is consistent with the experiment observation. The absence of LiOH and Li_2_O in the spectrum is attributed to their concentrations being below the detection limit. Given that the ester decomposition mechanism of the BA segment has been verified by both the XPS spectrum and AIMD simulation, we proposed that the BA segment of PAMPS-*co*-PBA can be tightly anchored to the Cu surface after the first Li plating by forming the ROLi. Meanwhile, the unreacted sulfo group of the AMPS segment orients outward from the surface, as observed in the simulation shown in Figure 7c. The well-oriented sulfo groups facilitate Li transportation at the interface and grant the self-healing capability of PAMPS-*co*-PBA, as illustrated in Figure 7d. Moreover, the short-chain lithium alkoxide resulting from the ester group decomposition forms the organic SEI components, which have high flexibility to accommodate the volume changes of the electrode during Li plating and stripping. Therefore, the PAMPS-*co*-PBA protected electrode exhibits a low Li nucleation overpotential and uniform surface morphology.

**Figure 8:**
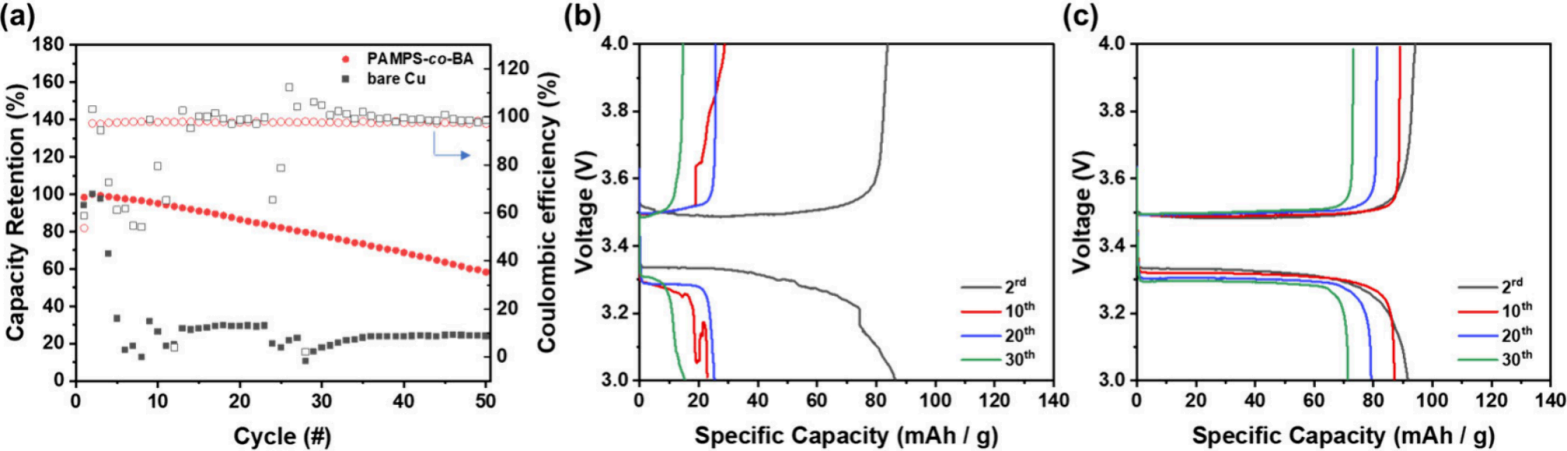
Electrochemical performance of anode-free LiFePO_4_ coin cells. (a) Cycling performance using PAMPS-*co*-PBA and Cu electrode with 1 M LiTFSI, DME/DOL (1/1, v/v), and 2 wt % LiNO_3_, with a potential range of 3.0 to 4.0 V and a C-rate of 0.1C. Voltage–capacity curves of (b) bare Cu/LFP and (c) PAMPS-*co*-PBA/LFP.

We tested the cycling of anode-free coin cells, comprising bare copper and copper with PAMPS-*co*-PBA interfaces paired with LiFePO_4_ as the cathode, in an ether electrolyte at a rate of 0.1 C. The results are shown in Figure 8a. The bare copper electrode shows rather poor stability, experiencing rapid fading during 50 cycles. The retention ratio was 35%, attributed to a fragile SEI and the absence of lithium dendrite suppression. In comparison, PAMPS-*co*-PBA electrode demonstrated 58.3% over 50 cycles which 1.6 times capacity retention than bare copper electrode. Figure 8b and c are voltage–capacity curves of bare Cu and PAMPS-*co*-PBA, respectively. The PAMPS-*co*-PBA electrode presents lower voltage polarization and capacity decay than the bare Cu electrode. The self-healing ability is evident in the restoration of artificial interface integrity without cracking, effectively preventing the consumption of Li-ions by parasitic reactions. Furthermore, the sulfonic acid component of AMPS is a single-ion conductor, contributing to the suppression of dendrite growth by minimizing concentration polarization. As a result, the PAMPS-*co*-PBA cell demonstrates much better capacity retention and lower voltage polarization compared with the bare copper cell. This can be attributed to minimized damage caused by the volume change during the cycle and the suppression of dendrite growth throughout cycling. However, it is noteworthy that the PAMPS-*co*-PBA cell exhibits capacity fading in each cycle, a phenomenon that possibly arises from the active Li loss. The TFSI was protonated by the sulfonic acid of AMPS segment, resulting in cleavage of the anion and capacity fading. This outcome suggests that the LiTFSI salt may not be a good choice for the PAMPS-*co*-PBA interface in future anode-free full cell applications.

Finally, to emphasize the advantages of PAMPS-*co*-PBA in stabilizing anode-free lithium metal batteries, we provide Table S7 containing a comparison of our work with other existing literature highlighting Coulombic efficiency and nucleation overpotential. Many previously reported artificial interfaces have inherent trade-offs in electrochemical performance.^18^ For instance, poly(vinylidene fluoride) (PVDF)/poly(methyl methacrylate) (PMMA) aims to maximize mechanical strength to prevent lithium fluctuation and dendrite-induced interface damage.^9^ However, interfaces lacking lithium-ion conductivity exhibit a higher nucleation barrier (94 mV), which can promote dendrite formation. Conversely, poly(ethylene oxide) (PEO)-based electrolytes achieve low nucleation overpotentials, facilitating smooth lithium deposition, but suffer from poor cycle life (∼40% after 100 cycles) due to continuous side reactions and SEI instability.^10^ To address these limitations, our group proposed the utilization of PAMPS-*co*-PBA which provides self-healing ability and single-ion conductivity and thus high potential to repair interfacial damage during volume fluctuation, enabling superior lithium metal stabilization. The combined effect of self-healing and single-ion conduction leads to enhanced lithium deposition, reduced dendrite growth, and extended battery life. In addition, the high performance of polyaryoxadiazole lithium sulfonate (PODLi), poly(vinylidene fluoride-hexafluoropropylene) (PVDF-HFP), and polyacrylonitrile (PAN) modifiers is due to the additional lithium salt applied in these systems. Notably, our approach achieves superior performance without the need for an external lithium source and effectively balances the aforementioned electrochemical trade-offs, demonstrating its effectiveness over other reported methods.

## Conclusion

The artificial interface of PAMPS-*co*-PBA with self-healing and single-ion conducting properties represents a promising approach toward anode-free lithium battery design, as evidenced by both experimental results and theoretical simulations. By overcoming the mechanical deficiency of AMPS through copolymerization with BA, the resulting interface achieves a balanced combination of softness, self-healing capabilities, and flat surface morphology, effectively mitigating degradation during lithium volume fluctuations. XPS analysis confirms the retention of self-healing and single-ion conductivity properties postcycling, leading to significantly improved performance in lithium plating and stripping tests. Additionally, DFT calculations elucidate the pathway of Li facilitated by the sulfo group, which acts to slow down dendrite formation. However, the decomposition of TFSI observed on the lithium surface in AIMD simulations indicates a potential risk to cyclic performance, suggesting the need for careful consideration of LiTFSI choice in a Lewis acid environment to further enhance electrochemical performance. In summary, our study proposes a creative design concept for an artificial interface aimed at suppressing dendrite formation in anode-free lithium batteries. By combining enhanced lithium conductivity, courtesy of the sulfo group, and self-healing properties to prevent SEI rupture, PAMPS-*co-*PBA emerges as a solution for enhancing the stability, efficiency, and cycle life of an anode-free battery through advanced interface engineering.

## Supporting Information Available

Supplementary Figures S1–S10. Experimental schematic of the PAMPS-*co*-PBA synthesis and electrode preparation; ^1^H NMR spectra of PAMPS-*co*-PBA, SEM cross-section image, EDS scanning for thickness analysis; Electrochemical performance evaluation of electrodes with different modified coatings; EIS result for interface checking; SEM and EDS mapping of the PBA electrode; AIMD simulation box and snapshots of the reaction of AMPS-BA and LiTFSI unit, LiTFSI away from the lithium metal interface. Supplementary Tables S1–S7. XPS deconvolution results and corresponding tables; Table of nucleation overpotential; Table of PAMPS-*co*-PBA and LiTFSI decomposition reactions during AIMD simulations; Table of literature for comparison (PDF)
