# Supplementary material for: Enhancing Anode-Free Battery Performance with Self-Healing Single-Ion Conducting PAMPS-co-PBA Copolymer Interfaces
Source: ACS Appl Mater Interfaces. 2025 Mar 18;17(16):23872–84. doi: 10.1021/acsami.4c22501 (PMC12022949; doi:10.1021/acsami.4c22501)
Supplement: Supplementary file 1 — am4c22501_si_001.pdf [file am4c22501_si_001.pdf]

Supporting information

Enhancing Anode-Free Battery Performance with Self-Healing Single-Ion Conducting PAMPS-*co*-PBA Copolymer Interfaces

Chia-Huan Chung[a #], Liang-Ting Wu[b #], Daniel Muara Sentosa[b], Chun-Chieh Ho[b], Po-Wei Chi[c d], Wen-Chia Hsu[e], Kuo-Wei Yeh[e], Chung-Chieh Chang[e], Bing Joe Hwang[b], Maw-Kuen Wu[c *], Jyh-Chiang Jiang[b *], Chien-Chieh Hu[a *] and Yu-Cheng Chiu[b *]

[a] Graduate Institute of Applied Science and Technology, National Taiwan University of Science and Technology, No. 43 Keelung Road, Sec 4, Taipei 10607, Taiwan

[b] Department of Chemical Engineering, National Taiwan University of Science and Technology, No. 43 Keelung Road, Sec 4, Taipei 10607, Taiwan

[c] Institute of Physics, Academia Sinica, No. 128, Section 2, Academia Road, Taipei 11529, Taiwan

[d] Department of Mechanical Engineering, Chung Yuan Christian University, No. 200, Chungpei Road, Chungli District, Taoyuan, 32023, Taiwan

[e] GUS Technology, Taoyuan, 32063, Taiwan

# contribution equally

*Corresponding author

Yu-Cheng Chiu : ycchiu@mail.ntust.edu.tw

Chien-Chieh Hu : cchu@mail.ntust.edu.tw

Jyh-Chiang Jiang : jcjiang@mail.ntust.edu.tw

Maw-Kuen Wu : mkwu@phys.sinica.edu.tw

Table of content

Synthesis of PAMPS-*co*-PBA S3

Copolymer concentration analysis using ^{1}H NMR S4

Electrode preparation and thickness characterization S5

Plating and stripping performance by PAMPS and PBA modification S5

Pre-Cyclic and cyclic modification interface resistance S6

PBA modification of copper after plating and stripping S7

Initial configuration of AIMD simulation box S8

Snapshots of the reaction during AIMD simulation S9

The XPS spectra of F 1s S9

The XPS of PAMPS-co-PBA electrode after 30th Li plating/stripping cycles, with and without methanol washing S10

The nucleation overpotential calculation S11

The decomposition reactions and reaction time during AIMD simulations S11

The XPS deconvolution analysis S12

Literatures comparing in artificial interface modify copper S16

References S17

Synthesis of PAMPS-*co*-PBA

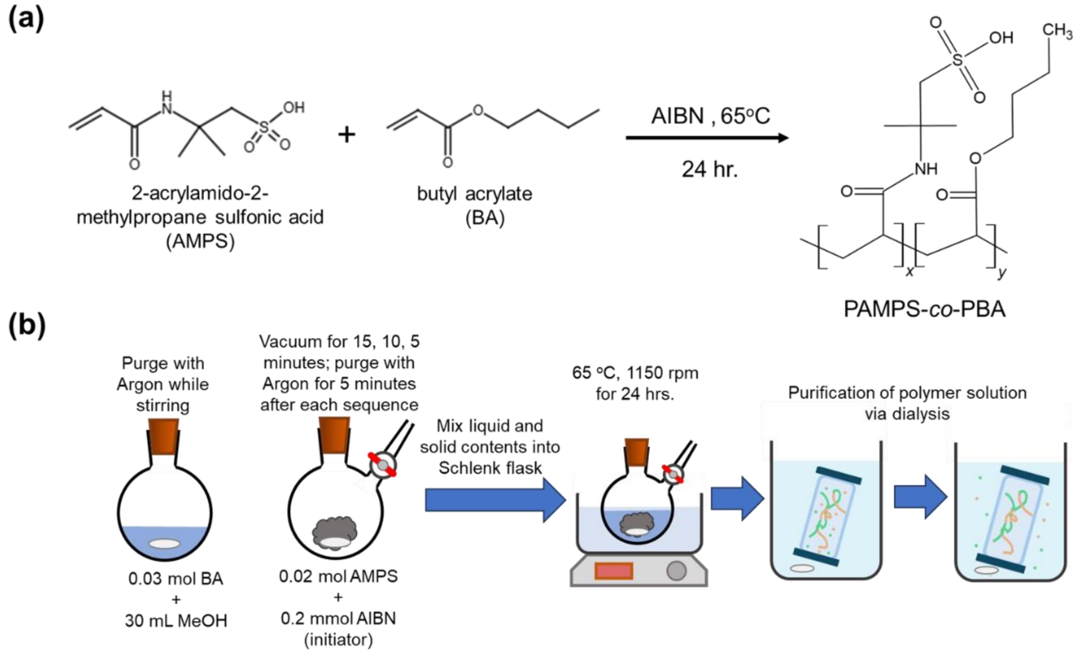

Figure S1. (a) Illustration of reaction process of PAMPS-*co*-PBA, and (b) the preparation process for PAMPS-*co*-PBA.

Copolymer concentration analysis using ^{1}HNMR

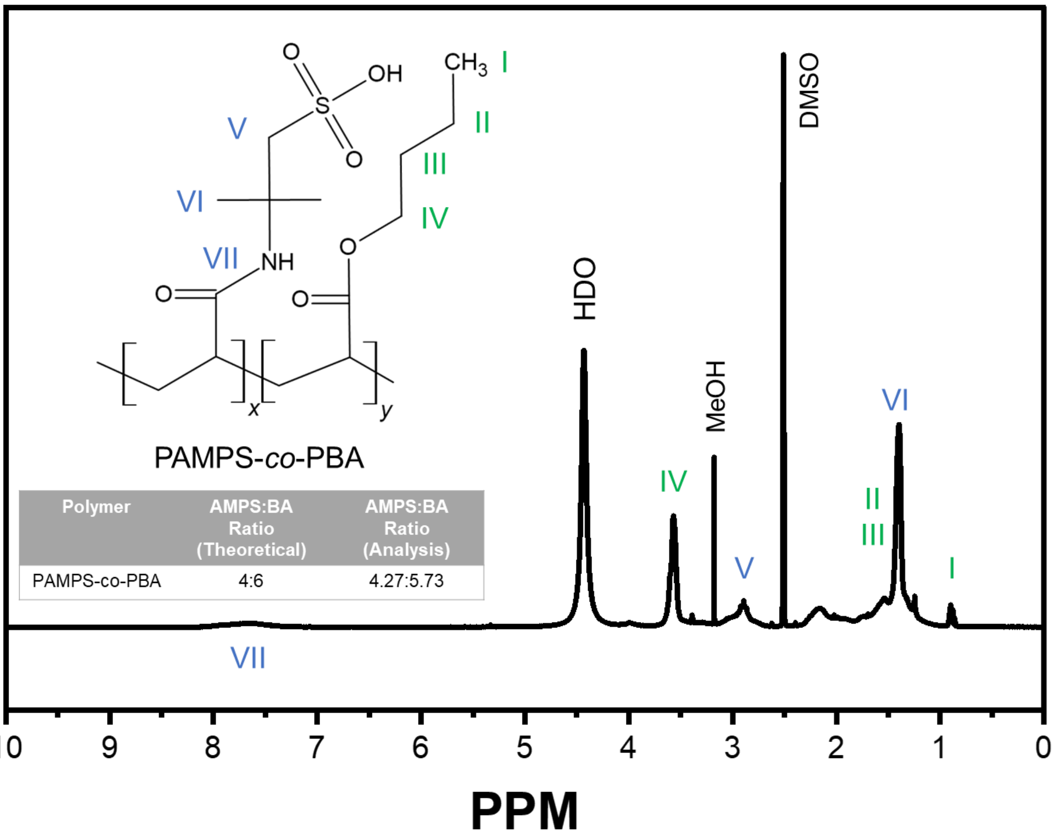

Polymer	AMPS:BA Ratio (Theoretical)	AMPS:BA Ratio (Analysis)
PAMPS-co-PBA	4:6	4.27:5.73

Figure S2. ^{1}H NMR spectra of PAMPS-*co*-PBA.

Electrode preparation and thickness characterization

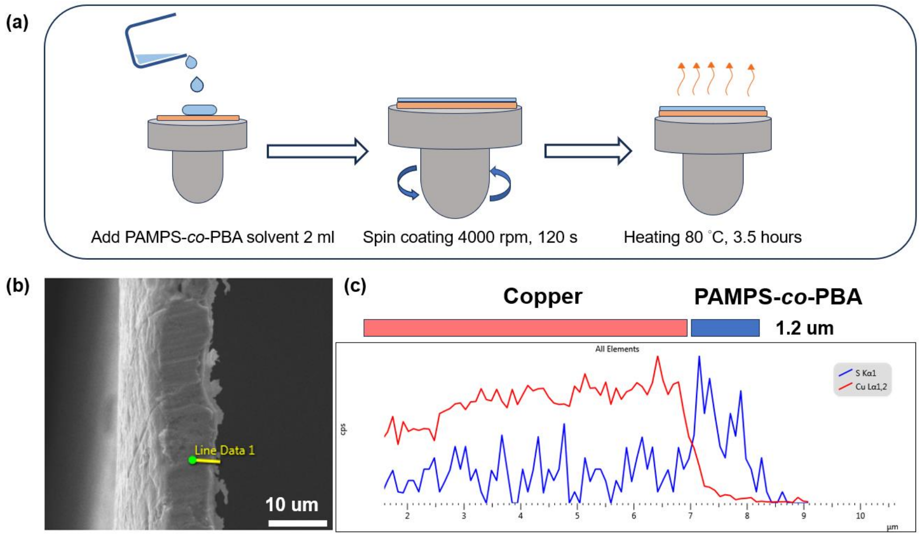

Figure S3. The electrode preparation method, SEM cross section and EDS line scanning (a) Schematic diagram of PAMPS-*co*-PBA electrode prepared on copper. (b) The thickness present by SEM cross section. (c) EDS line scanning.

Plating and stripping performance by PAMPS and PBA modification

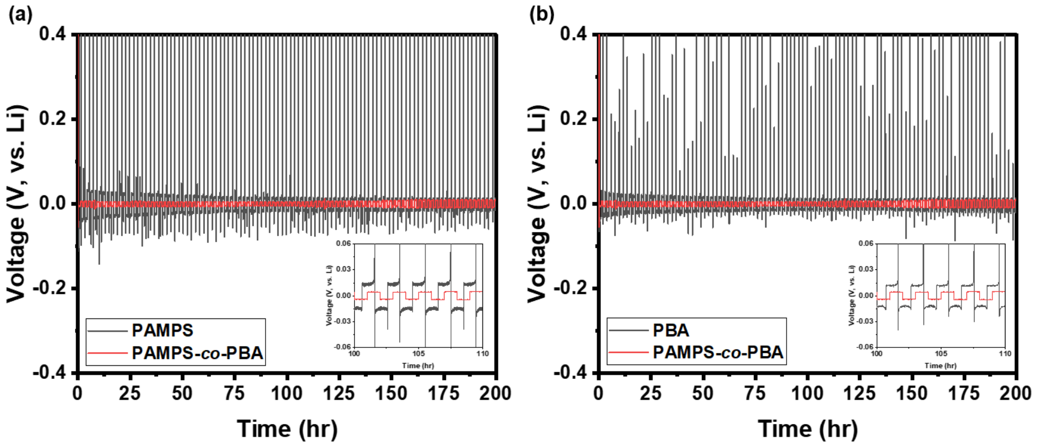

Figure S4. Electrochemical performance comparison of Li/Cu half cells with modified coating. (a) Cycle stability comparison of PAMPS and PAMPS-*co*-PBA, both at a deposition capacity and current density of 0.5 mA cm^{-2} and 0.5 mAh cm^{-2}, respectively(b) Cycle stability comparison of PAMPS and PAMPS-*co*-PBA, both at a deposition capacity and current density of 0.5 mA cm^{-2} and 0.5 mAh cm^{-2}, respectively.

Pre-Cyclic and cyclic modification interface resistance

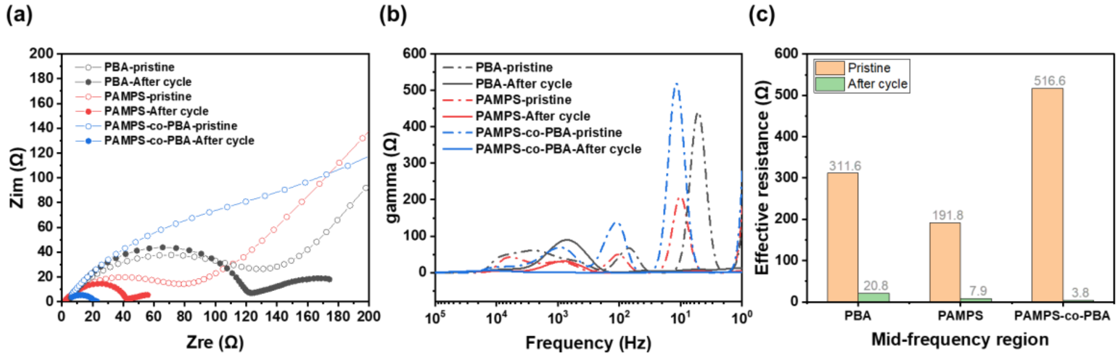

Figure S5. (a) EIS analysis and (b) DRT calculations of the pristine and 30th-cycle coin cell. (c) Effective resistances extracted from the DRT results. The plating and stripping test was conducted at a current density of 0.5 mA cm^{-2} and a capacity of 0.5 mAh cm^{-2} using an electrolyte composed of 1 M LiTFSI in DME/DOL (1/1, v/v) with 2 wt% $LiNO_3$.

PBA modification of copper after plating and stripping

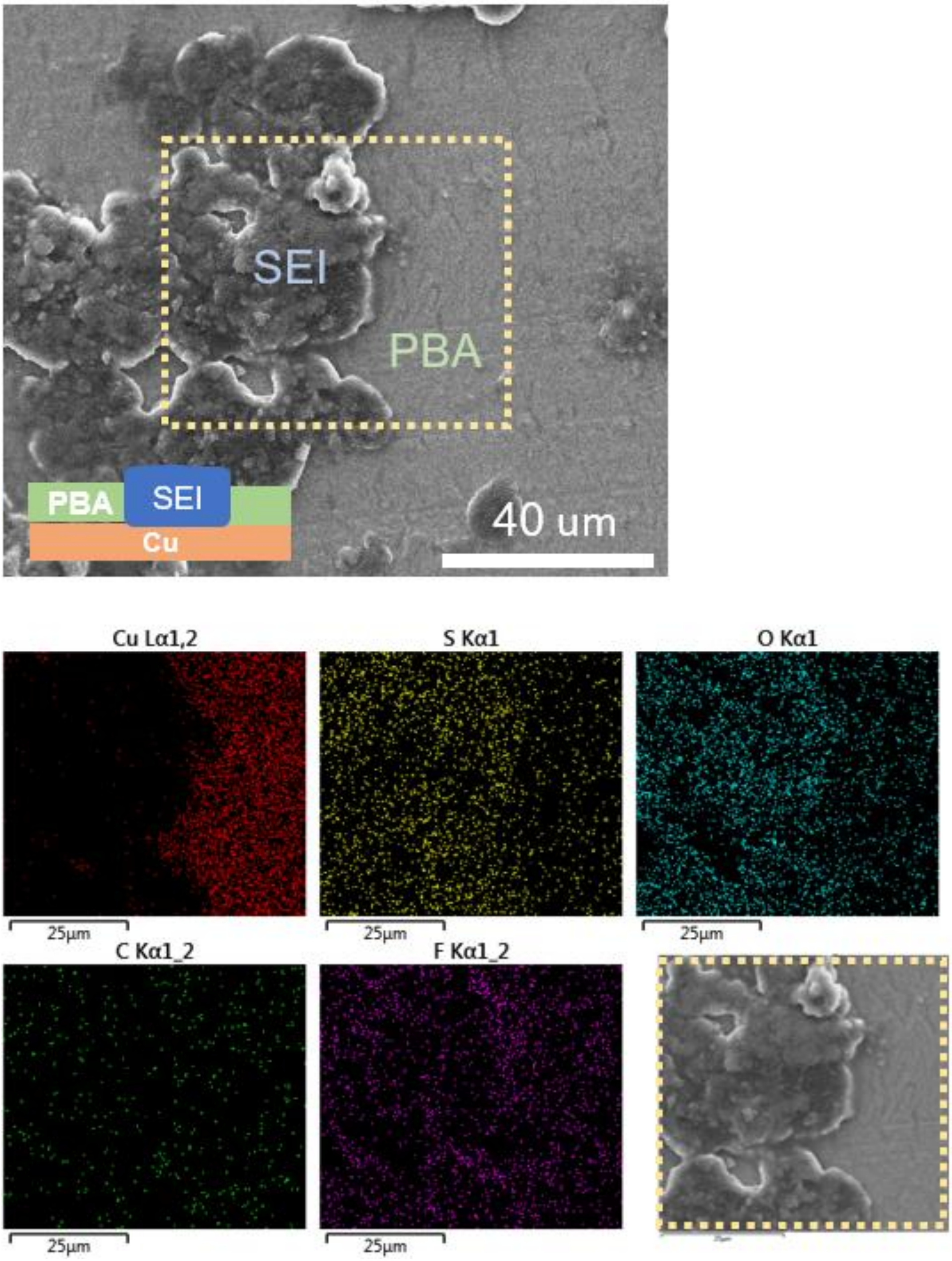

Figure S6. The SEM and EDS mapping of PBA electrode surface after 100th cycle at a deposition capacity and current density of 0.5 mA cm^{-2} and 0.5 mAh cm^{-2} using an electrolyte composed of 1 M LiTFSI in DME/DOL (1/1, v/v) with 2 wt% $LiNO_3$.

Initial configuration of AIMD simulation box

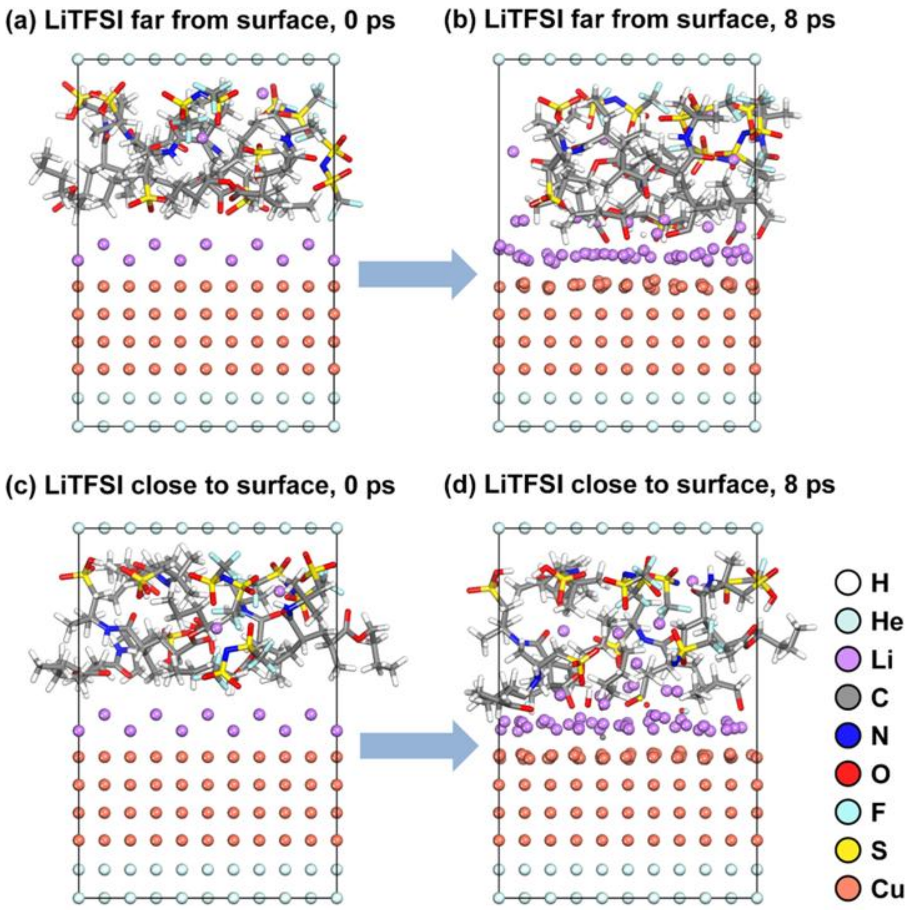

Figure S7. AIMD simulation box for PAMPS-*co*-PBA on Lithium (100) surface. Two environments were calculated (a) LiTFSI far from lithium metal and (c) LiTFSI close to lithium metal, both at 0 ps,. (b and d) present the end states of AIMD simulation at 8 ps.

Snapshots of the reaction during AIMD simulation

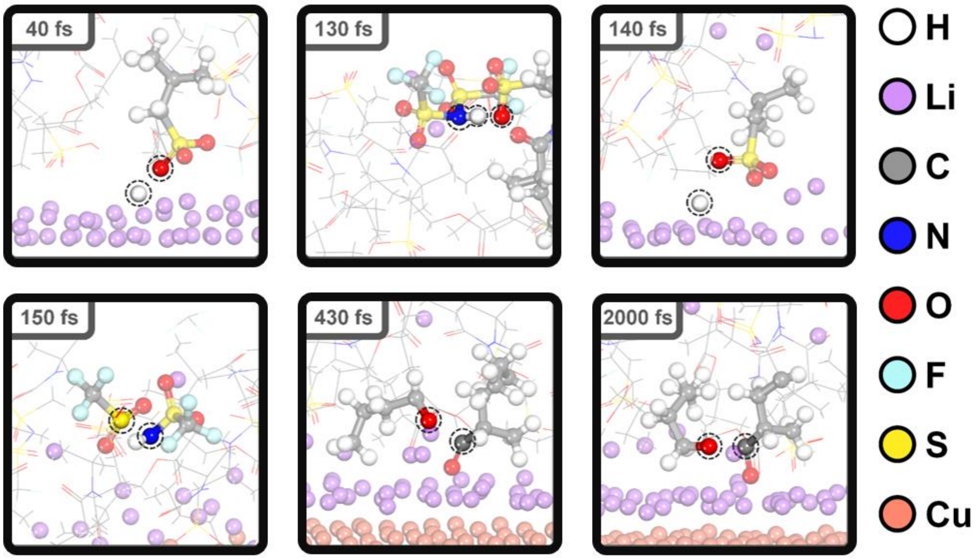

Figure S8. Snapshots of the reaction of PAMPS-*co*-PBA and LiTFSI unit, LiTFSI away from the lithium metal interface.

The XPS spectra of F 1s

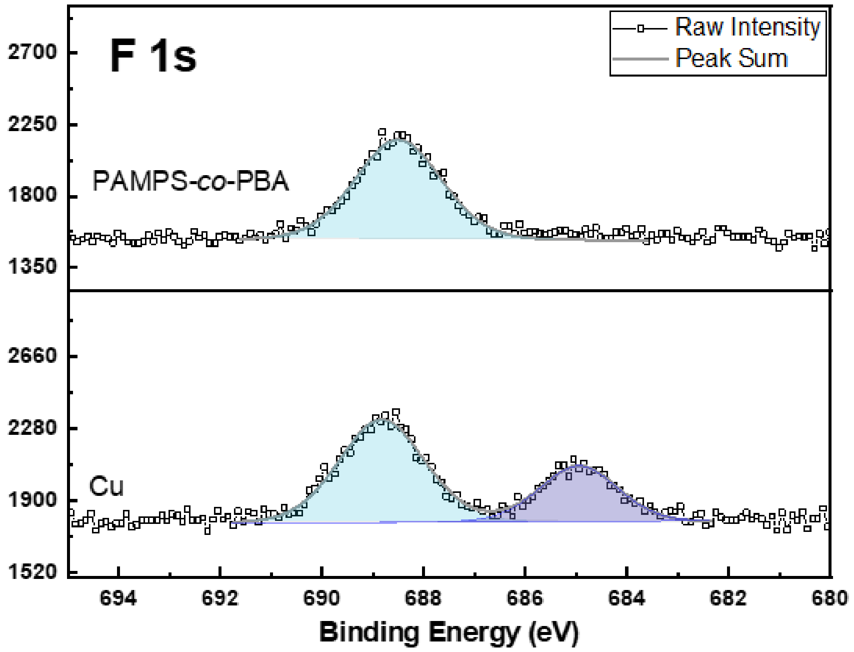

Figure S9. XPS spectra of F 1s for the bare copper and PAMPS-*co*-PBA interface after one cycle of Li plating and stripping processes.

The XPS of PAMPS-co-PBA electrode after 30th Li plating/stripping cycles, with and without methanol washing.

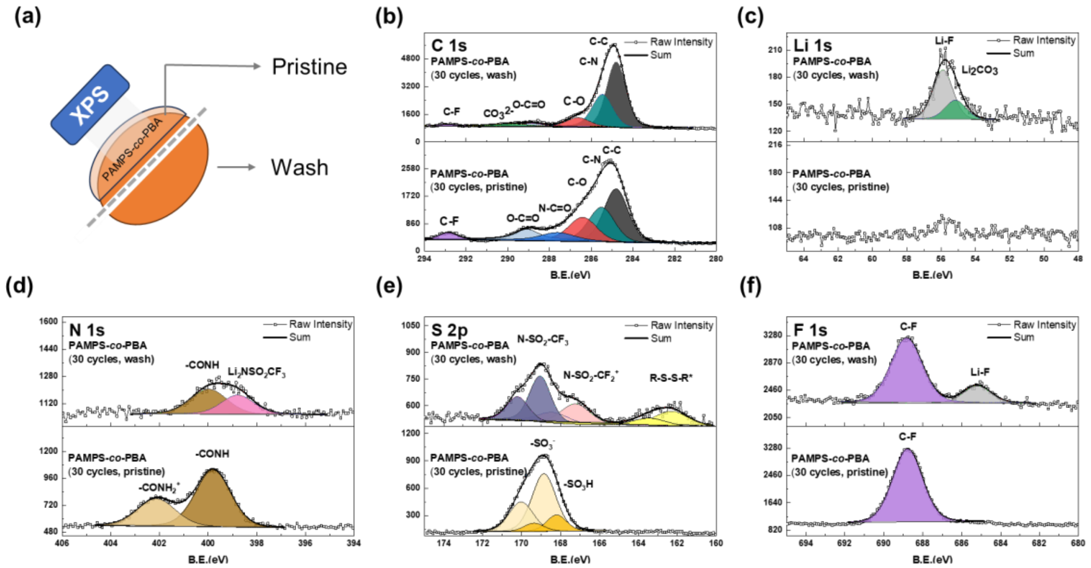

Figure S10. Comparison PAMPS-*co*-PBA-modified copper surface after 30th lithium plating/stripping cycles, with and without methanol washing. (a) Schematic illustration of experiment. XPS spectra of (b)C 1s, (c) Li 1s, (d) N 1s, (e) S 2p, and (f) F 1s.

The nucleation overpotential calculation

Table S1. The value of bare Cu and modified Cu during the first cycle discharge process in voltage-capacity curve**.**

**(unit : mV)**	**Bottom voltage (Nucleation overpotential +mass-transfer overpotential)**	**Platform voltage (mass-transfer overpotential)**	**Nucleation overpotential**
**bare Cu**	235	103	132
**PBA**	179	76	103
**PAMPS**	158	84	74
**PAMPS-*co*-PBA**	127	69	58

The decomposition reactions and reaction time during AIMD simulations

Table S2. Catalog of PAMPS-*co*-PBA and LiTFSI decomposition reactions and approximate occurring time during AIMD simulations when LiTFSI is far from lithium surface.

Species	Time (fs)	Reaction
PAMPS-*co*-PBA	40	$R\text{-}SO_3H + Li \rightarrow R\text{-}SO_3 + H + Li$
PAMPS-*co*-PBA、TFSI	130	$N(SO_2CF_3)_2 + R\text{-}SO_3H \rightarrow HN(SO_2CF_3)_2 + R\text{-}SO_3$
PAMPS-*co*-PBA	140	$R\text{-}SO_3H \rightarrow R\text{-}SO_3 + H$
TFSI	150	$HN(SO_2CF_3)_2 \rightarrow HNSO_2CF_3 + SO_2CF_3$
PAMPS-*co*-PBA	430	R(C=O)OR'→ R(C=O) + R'-O
PAMPS-*co*-PBA	2000	R(C=O)OR'→ R(C=O) + R'-O

The XPS deconvolution analysis

Table S3. The XPS deconvolution to binding energies, FWHM and Area, and observed attribution of species via XPS in bare copper surface after first cycle plating/stripping.

**Detail spectrum**	**Binding energy [eV]**	**Attributed species**	**FWHM**	**Area**	**Ref**	**Assumed Origin**
**C 1s**	284.8	C-C	1.31	7561.99	[1,2]	DME/DOL
	285.6	C-N	1.37	1529.92	[3]	LiTFSI
	286.7	C-O	1.42	1273.35	[2,4–6]	DOL
	288.9	O-C=O	1.5	827.51	[7–9]	DME/DOL
	290.2	CO_3^{2-}	1.19	1009.86	[2,4,5,8]	Carbonates, e.g., Li_2CO_3 or semicarbonates
	290.9	CF_2	1.3	182.02	[4,7,10]	LiTFSI
**F 1s**	685.0	LiF	1.82	630.32	[2,4,11,12]	LiTFSI
	688.9	CF	1.99	1268.29	[2,5,12,13]	LiTFSI
**Li 1s**	55.3	Li_2CO_3	1.63	258.13	[5,14]	DME/DOL
	55.8	LiF	1.84	160.06	[1,10]	LiTFSI
**N 1s**	399.36	$N\text{-}SO_2$	2.55	199.62	[2,15]	LiTFSI
**O 1s**	531.1	ROLi	1.77	4683.50	[16,17]	DME/DOL
	532.0	$N\text{-}SO_2$	1.51	8971.46	[8]	LiTFSI
	532.7	C=O	2.07	2449.26	[18]	DME/DOL/ LiTFSI
	533.6	C-O	1.62	2027.95	[18]	DME/DOL/ LiTFSI
**S $2p_{3/2}$**	167.4	$N\text{-}SO_2$ or $N\text{-}SO_2^-$ CF_2^+	1.48	55.65	[2,10,15]	LiTFSI
	169.2	$N\text{-}SO_2\text{-}CF_3$	1.25	149.13	[2,15]	LiTFSI

Table S4. The XPS deconvolution to binding energies, FWHM, area, and observed attribution of species via XPS in PAMPS-*co*-PBA modification surface after first cycle plating/stripping.

Detail spectrum	Binding energy [eV]	Attributed species	FWHM	Area	Ref	Assumed Origin
**C 1s**	284.8	C-C	1.26	7547.75	[1,2]	DME/DOL or PAMPS-*co*-PBA
	285.6	C-N	1.21	2825.79	[3]	LiTFSI
	286.6	C-O	1.21	2143.50	[2,4–6]	PAMPS-*co*-PBA
	287.8	N-C=O	1.11	369.91	[9,19]	PAMPS-*co*-PBA
	289.0	O-C=O	1.14	1269.21	[7–9]	PAMPS-*co*-PBA
**F 1s**	688.6	C-F	1.87	1367.11	[2,5,12,13]	LiTFSI
**Li 1s**	ND					
**N 1s**	399.8	CONH	1.57	993.61	[6,9]	PAMPS-*co*-PBA
	401.6	$CONH^{2+}$	2.36	426.96	[6]	PAMPS-*co*-PBA
**O 1s**	531.4	$-SO_3H$	1.26	3010.58	[20]	PAMPS-*co*-PBA
	532.1	$N-SO_2$	1.09	4431.94	[8]	LiTFSI
	532.8	C=O	1.12	2392.15	[18]	DME/DOL/ LiTFSI
	533.7	C-O	1.49	3876.89	[18]	DME/DOL/ LiTFSI
**S $2p_{3/2}$**	168.1	$-SO_3H$	1.2	757.32	[9]	PAMPS-*co*-PBA
	168.9	$-SO_{3-}$	1.34	524.49	[21]	PAMPS-*co*-PBA

Table S5. The XPS deconvolution to binding energies, FWHM, area, and observed attribution of species via XPS in PAMPS-*co*-PBA modification surface after 30 cycles plating/stripping.

Detail spectrum	Binding energy [eV]	Attributed species	FWHM	Area	Ref	Assumed Origin
**C 1s**	284.8	C-C	1.37	2702.42	[1,2]	DME/DOL/ PAMPS-*co*-PBA
	285.5	C-N	1.47	1824.57	[3]	LiTFSI
	286.4	C-O	1.55	1315.34	[2,4–6]	PAMPS-*co*-PBA
	287.6	N-C=O	1.79	480.11	[9,19]	PAMPS-*co*-PBA
	289.0	O-C=O	1.35	642.46	[7–9]	PAMPS-*co*-PBA
	292.9	CF_3	1	242.91	[2,7,8,12]	LiTFSI
**F 1s**	688.8	CF	1.82	4712.67	[2,5,12,13]	LiTFSI
**Li 1s**	ND					
**N 1s**	399.8	CONH	1.72	1029.94	[6,9]	PAMPS-*co*-PBA
	402.1	$CONH_2^+$	1.83	521.30	[6]	PAMPS-*co*-PBA
**O 1s**	531.5	$-SO_3H$	1.27	1846.17	[20]	PAMPS-*co*-PBA
	532.2	$N-SO_2$	1.12	3183.67	[8]	LiTFSI
	532.9	C=O	1.07	2261.32	[18]	DME/DOL/ LiTFSI
	533.8	C-O	1.58	2121.29	[18]	DME/DOL/ LiTFSI
**S 2p3/2**	168.2	$-SO_3H$	1.47	1092.50	[9]	PAMPS-*co*-PBA
	168.9	$-SO_3^-$	1.2	254.82	[21]	PAMPS-*co*-PBA

Table S6. The XPS deconvolution to binding energies, FWHM, area, and observed attribution of species via XPS in PAMPS-*co*-PBA modification surface after 30 cycles plating/stripping and wash with methanol.

Detail spectrum	Binding energy [eV]	Attributed species	FWHM	Area	Ref	Assumed Origin
**C 1s**	284.8	C-C	1.07	4722.03	1,2	DME/DOL/ PAMPS-co-PBA
	285.5	C-N	1.17	2542.89	3	LiTFSI
	286.6	C-O	1.28	739.10	2,4–6	DME/DOL/ PAMPS-co-PBA
	288.9	O-C=O	1.24	223.59	7–9	DME/DOL/ PAMPS-co-PBA
	289.9	CO_3^{2-}	1.83	251.16	2,4,5,8	Carbonates, e.g., Li_2CO_3 or semicarbonates
	292.9	CF_3	0.97	120.67	2,7,8,12	LiTFSI
**F 1s**	685.2	LiF	1.67	515.95	2,4,11,12	LiTFSI
	688.8	CF	1.92	2188.24	2,5,12,13	LiTFSI
**Li 1s**	55.1	Li_2CO_3	1.43	36.39	5,14	DME/DOL/ LiTFSI
	55.9	LiF	1.37	88.78	1,10	LiTFSI
**N 1s**	398.8	$Li_2NSO_2CF_3$	1.58	207.74	15,22	LiTFSI
	400.0	CONH	1.77	305.24	6,9	PAMPS-co-PBA
**O 1s**	531.2	ROLi	1.59	891.35	16	DME/DOL
	532.0	$N\text{-}SO_2$	1.23	2752.02	8	LiTFSI
	532.8	C=O	1.32	2178.6	18	DME/DOL/ LiTFSI
	533.8	C-O	1.44	1280.68	18	DME/DOL/ LiTFSI
**S 2p3/2**	162.3	R-S-S-R’	1.79	240.07	23	Derivative form DME/DOL/ LiTFSI
	167.3	NSO2 or $NSO_2CF_2^+$	1.29	386.60	2,10,15	LiTFSI
	169.1	NSO_2CF_3	2.03	194.63	2,15	LiTFSI

Literatures comparing in artificial interface modify copper

Table S7. Comparison table of various artificial interface covering copper surface, resulting in plating/stripping coulombic efficiency, nucleation overpotential and retention capacity.

**Polymer**	**Electrolyte**	**Subtract**	**Plating/Stripping Coulombic Efficiency (%)**	**Nucleation Overpotential (mV)**	**Retention Capacity**	**Ref**
PAMPS-*co*-PBA	1M LiTFSI DME/DOL 2% $LiNO_3$	Cu	100% (100th, 0.5 mA cm^{-2})	58	58.3% (50th, LFP)	This work
PVDF/PMMA	1M LiTFSI DME/DOL 2% $LiNO_3$	Cu	98.3% (260th, 1 mA cm^{-2})	94	92% (200th, LFP)*	24
PEO	1M LiTFSI DME/DOL 2% $LiNO_3$	Cu	~100% (200th, 0.5 mA cm^{-2})	32	40.5% (100th, LFP)	25
PODLi	1M LiTFSI DME/DOL 2% $LiNO_3$	Cu	98.5% (500th ,1 mA cm^{-2})	9	81% (200th,LFP)	26
PVDF-HFP	3M LiFSI EC/DMC	Cu	99.15% (200th ,0.5 mA cm^{-2})	10*	40% (80th, NCM111)	27
PAN	1M LiTFSI DME/DOL 2% $LiNO_3$	Cu	98.2% (250th, 0.5 mA cm^{-2})	160	90% (100th, LFP, pre-deposited Li on Cu)	28
BC-*g*-P(EGM-*co*-TFEA)	1M $LiPF_6$ EC/DCE	Cu	98.6% (120th, 1 mA cm^{-2})	21	92% (50th, LFP)*	29

*Due to the lack of tabulated data, the analytical result value was determined based on ImageJ estimates of the graphical data presented in the reference journal.

PVDF= poly(vinylidene fluoride), PMMA = poly(methyl methacrylate), PEO = Poly(ethylene oxide), PODLi = polyaryoxadiazole lithium sulfonate, PVDF-HFP = poly (vinylidene fluoride-hexafluoropropylene), PAN = polyacrylonitrile, BC = bacterial cellulose, PEGM = poly(diethylene glycol methyl ether methacrylate, PTFEA= poly(2,2,2-trifluoroethyl acrylate).

References

(1) Hu, Z.; Zhang, S.; Dong, S.; Li, Q.; Cui, G.; Chen, L. Self-Stabilized Solid Electrolyte Interface on a Host-Free Li-Metal Anode toward High Areal Capacity and Rate Utilization. *Chem. Mater.* **2018**, *30* (12), 4039–4047. https://doi.org/10.1021/acs.chemmater.8b00722.

(2) Busche, M. R.; Weiss, M.; Leichtweiss, T.; Fiedler, C.; Drossel, T.; Geiss, M.; Kronenberger, A.; Weber, D. A.; Janek, J. The Formation of the Solid/Liquid Electrolyte Interphase (SLEI) on NASICON-Type Glass Ceramics and LiPON. *Adv. Mater. Interfaces* **2020**, *7* (19), 2000380. https://doi.org/10.1002/admi.202000380.

(3) Chu, H.; Noh, H.; Kim, Y.-J.; Yuk, S.; Lee, J.-H.; Lee, J.; Kwack, H.; Kim, Y.; Yang, D.-K.; Kim, H.-T. Achieving Three-Dimensional Lithium Sulfide Growth in Lithium-Sulfur Batteries Using High-Donor-Number Anions. *Nat. Commun.* **2019**, *10* (1), 188. https://doi.org/10.1038/s41467-018-07975-4.

(4) Leroy, S.; Martinez, H.; Dedryvère, R.; Lemordant, D.; Gonbeau, D. Influence of the Lithium Salt Nature over the Surface Film Formation on a Graphite Electrode in Li-Ion Batteries: An XPS Study. *Appl. Surf. Sci.* **2007**, *253* (11), 4895–4905. https://doi.org/10.1016/j.apsusc.2006.10.071.

(5) Ismail, I.; Noda, A.; Nishimoto, A.; Watanabe, M. XPS Study of Lithium Surface after Contact with Lithium-Salt Doped Polymer Electrolytes. *Electrochimica Acta* **2001**, *46* (10–11), 1595–1603. https://doi.org/10.1016/S0013-4686(00)00758-1.

(6) Zhang, C.; Easteal, A. J. Thermoanalytical, Spectroscopic, and Morphological Study of Poly(Ethylene Glycol)/Poly(2-acrylamido-2-methylpropanesulfonic Acid- *Co* - *N* -isopropylacrylamide) Semi-interpenetrating Network Gels. *J. Appl. Polym. Sci.* **2007**, *104* (3), 1723–1731. https://doi.org/10.1002/app.25812.

(7) Alvarado, J.; Schroeder, M. A.; Pollard, T. P.; Wang, X.; Lee, J. Z.; Zhang, M.; Wynn, T.; Ding, M.; Borodin, O.; Meng, Y. S.; Xu, K. Bisalt Ether Electrolytes: A Pathway towards Lithium Metal Batteries with Ni-Rich Cathodes. *Energy Environ. Sci.* **2019**, *12* (2), 780–794. https://doi.org/10.1039/C8EE02601G.

(8) Andersson, E. K. W.; Sångeland, C.; Berggren, E.; Johansson, F. O. L.; Kühn, D.; Lindblad, A.; Mindemark, J.; Hahlin, M. Early-Stage Decomposition of Solid Polymer Electrolytes in Li-Metal Batteries. *J. Mater. Chem. A* **2021**, *9* (39), 22462–22471. https://doi.org/10.1039/D1TA05015J.

(9) Kalinov, K. N.; Ignatova, M. G.; Manolova, N. E.; Markova, N. D.; Karashanova, D. B.; Rashkov, I. B. Novel Antibacterial Electrospun Materials

Based on Polyelectrolyte Complexes of a Quaternized Chitosan Derivative. *RSC Adv.* **2015**, *5* (67), 54517–54526. https://doi.org/10.1039/C5RA08484A.
(10) Alvarado, J.; Schroeder, M. A.; Zhang, M.; Borodin, O.; Gobrogge, E.; Olguin, M.; Ding, M. S.; Gobet, M.; Greenbaum, S.; Meng, Y. S.; Xu, K. A Carbonate-Free, Sulfone-Based Electrolyte for High-Voltage Li-Ion Batteries. *Mater. Today* **2018**, *21* (4), 341–353. https://doi.org/10.1016/j.mattod.2018.02.005.
(11) Fang, J.; Li, J.; Qin, L.; Li, A.; Feng, H. Atomic/Molecular Layer-Deposited Laminated Li_2O–Lithicone Interfaces Enabling High-Performance Silicon Anodes. *ACS Appl. Mater. Interfaces* **2023**, *15* (23), 27963–27974. https://doi.org/10.1021/acsami.3c02925.
(12) Xu, H.; Chien, P.-H.; Shi, J.; Li, Y.; Wu, N.; Liu, Y.; Hu, Y.-Y.; Goodenough, J. B. High-Performance All-Solid-State Batteries Enabled by Salt Bonding to Perovskite in Poly(Ethylene Oxide). *Proc. Natl. Acad. Sci.* **2019**, *116* (38), 18815–18821. https://doi.org/10.1073/pnas.1907507116.
(13) Sharova, V.; Moretti, A.; Diemant, T.; Varzi, A.; Behm, R. J.; Passerini, S. Comparative Study of Imide-Based Li Salts as Electrolyte Additives for Li-Ion Batteries. *J. Power Sources* **2018**, *375*, 43–52. https://doi.org/10.1016/j.jpowsour.2017.11.045.
(14) Hornsveld, N.; Put, B.; Kessels, W. M. M.; Vereecken, P. M.; Creatore, M. Plasma-Assisted and Thermal Atomic Layer Deposition of Electrochemically Active Li_2CO_3. *RSC Adv.* **2017**, *7* (66), 41359–41368. https://doi.org/10.1039/C7RA07722J.
(15) Forster-Tonigold, K.; Buchner, F.; Bansmann, J.; Behm, R. J.; Groß, A. A Combined XPS and Computational Study of the Chemical Reduction of BMP-TFSI by Lithium**. *Batter. Supercaps* **2022**, *5* (12), e202200307. https://doi.org/10.1002/batt.202200307.
(16) Li, Q.; Wang, Y.; Wang, X.; Sun, X.; Zhang, J.-N.; Yu, X.; Li, H. Investigations on the Fundamental Process of Cathode Electrolyte Interphase Formation and Evolution of High-Voltage Cathodes. *ACS Appl. Mater. Interfaces* **2020**, *12* (2), 2319–2326. https://doi.org/10.1021/acsami.9b16727.
(17) Tatara, R.; Karayaylali, P.; Yu, Y.; Zhang, Y.; Giordano, L.; Maglia, F.; Jung, R.; Schmidt, J. P.; Lund, I.; Shao-Horn, Y. The Effect of Electrode-Electrolyte Interface on the Electrochemical Impedance Spectra for Positive Electrode in Li-Ion Battery. *J. Electrochem. Soc.* **2019**, *166* (3), A5090–A5098. https://doi.org/10.1149/2.0121903jes.
(18) Guo, C.; Wu, Y.; Li, Z.; Liao, W.; Sun, L.; Wang, C.; Wen, B.; Li, Y.; Chen, C. The Oxygen Reduction Electrocatalytic Activity of Cobalt and Nitrogen Co-Doped Carbon Nanocatalyst Synthesized by a Flat Template. *Nanoscale Res.*

Lett. **2017**, *12* (1), 144. https://doi.org/10.1186/s11671-016-1804-z.
(19) Xiao, C.; Lin, J. PAMPS- *Graft* -$Ni_3Si_2O_5(OH)_4$ Multiwalled Nanotubes as a Novel Nano-Sorbent for the Effective Removal of Pb(II) Ions. *RSC Adv.* **2020**, *10* (13), 7619–7627. https://doi.org/10.1039/C9RA10971D.
(20) Leroux, F.; Illaik, A.; Stimpfling, T.; Troutier-Thuilliez, A.-L.; Fleutot, S.; Martinez, H.; Cellier, J.; Verney, V. Percolation Network of Organo-Modified Layered Double Hydroxide Platelets into Polystyrene Showing Enhanced Rheological and Dielectric Behavior. *J. Mater. Chem.* **2010**, *20* (42), 9484. https://doi.org/10.1039/b926978a.
(21) Mandal, S.; Kumari, S.; Kumar, M.; Ojha, U. Supplementary Networking of Interpenetrating Polymer System (SNIPSy) Strategy to Develop Strong & High Water Content Ionic Hydrogels for Solid Electrolyte Applications. *Adv. Funct. Mater.* **2021**, *31* (26), 2100251. https://doi.org/10.1002/adfm.202100251.
(22) Donzelli, M.; Ferber, T.; Vanita, V.; Waidha, A. I.; Müller, P.; Mellin, M.; Hausbrand, R.; Jaegermann, W.; Clemens, O. On the Surface Modification of LLZTO with LiF via a Gas-Phase Approach and the Characterization of the Interfaces of LiF with LLZTO as Well as PEO+LiTFSI. *Materials* **2022**, *15* (19), 6900. https://doi.org/10.3390/ma15196900.
(23) Zhang, Q.; Chen, J.-J.; Wang, X.-Y.; Yang, C.; Zheng, M.-S.; Dong, Q.-F. Enhanced Electrochemical Performance and Thermal Stability of $LiNi_{0.5}Mn_{1.5}O_4$ Using an Electrolyte with Sulfolane. *Phys. Chem. Chem. Phys.* **2015**, *17* (16), 10353–10357. https://doi.org/10.1039/C5CP00799B.
(24) Xiong, X.; Zhi, R.; Zhou, Q.; Yan, W.; Zhu, Y.; Chen, Y.; Fu, L.; Yu, N.; Wu, Y. A Binary PMMA/PVDF Blend Film Modified Substrate Enables a Superior Lithium Metal Anode for Lithium Batteries. *Mater. Adv.* **2021**, *2* (13), 4240–4245. https://doi.org/10.1039/D1MA00121C.
(25) Assegie, A. A.; Cheng, J.-H.; Kuo, L.-M.; Su, W.-N.; Hwang, B.-J. Polyethylene Oxide Film Coating Enhances Lithium Cycling Efficiency of an Anode-Free Lithium-Metal Battery. *Nanoscale* **2018**, *10* (13), 6125–6138. https://doi.org/10.1039/C7NR09058G.
(26) Yu, Y.; Yang, C.; Jiang, Y.; Zhu, J.; Zhang, J.; Jiang, M. An Efficient Protective Layer with High Ionic Conductivity Enables Long-Life and Dendrite-Free Li Metal Anodes. *CCS Chem.* **2024**, *0* (0), 1–11. https://doi.org/10.31635/ccschem.024.202404524.
(27) Nikodimos, Y.; Su, W.-N.; Shitaw, K. N.; Jiang, S.-K.; Abrha, L. H.; Weret, M. A.; Merso, S. K.; Hagos, T. M.; Huang, C.-J.; Lakshmanan, K.; Huang, W.-H.; Chang, C.-Y.; Lin, J.-M.; Wu, S.-H.; Yang, C.-C.; Hwang, B. J. Multifunctional Electrospun PVDF-HFP Gel Polymer Electrolyte Membrane Suppresses

Dendrite Growth in Anode-Free Li Metal Battery. *Energy Storage Mater.* **2023**, *61*, 102861. https://doi.org/10.1016/j.ensm.2023.102861.

(28) Liu, Y.; Xu, Y.; Wang, J.; Sun, Y.; Feng, X.; Xiang, H. Regulated Lithium Deposition Behavior by an Artificial Coating of Cu Foil for Dendrite-Free Lithium Metal Batteries. *Mater. Today Sustain.* **2022**, *18*, 100127. https://doi.org/10.1016/j.mtsust.2022.100127.

(29) Liu, R.; Song, S.; Xue, J.; Deng, H.; Li, S.; Cheng, Q. A Robust Protective Layer Based on Polymer Brush for Long-Term Cycling of Li Metal Anodes. *ACS Appl. Energy Mater.* **2024**, *7* (22), 10605–10613. https://doi.org/10.1021/acsaem.4c02150.
